# A Review and Meta-Analysis of Influenza Interactome Studies

**DOI:** 10.3389/fmicb.2022.869406

**Published:** 2022-04-21

**Authors:** Sonja Courtney Jun Hui Chua, Jianzhou Cui, David Engelberg, Lina Hsiu Kim Lim

**Affiliations:** ^1^Department of Physiology, Yong Loo Lin School of Medicine, National University of Singapore, Singapore, Singapore; ^2^Immunology Translational Research Program, Yong Loo Lin School of Medicine, National University of Singapore, Singapore, Singapore; ^3^NUS Immunology Program, Life Sciences Institute, National University of Singapore, Singapore, Singapore; ^4^CREATE-NUS-HUJ Cellular & Molecular Mechanisms of Inflammation Programme, National University of Singapore, Singapore, Singapore; ^5^Department of Microbiology, Yong Loo Lin School of Medicine, National University of Singapore, Singapore, Singapore; ^6^Department of Biological Chemistry, The Institute of Life Science, The Hebrew University of Jerusalem, Jerusalem, Israel

**Keywords:** influenza, host-pathogen interactions, influenza proteins, interactome analysis, bioinformatics

## Abstract

Annually, the influenza virus causes 500,000 deaths worldwide. Influenza-associated mortality and morbidity is especially high among the elderly, children, and patients with chronic diseases. While there are antivirals available against influenza, such as neuraminidase inhibitors and adamantanes, there is growing resistance against these drugs. Thus, there is a need for novel antivirals for resistant influenza strains. Host-directed therapies are a potential strategy for influenza as host processes are conserved and are less prone mutations as compared to virus-directed therapies. A literature search was performed for papers that performed viral–host interaction screens and the Reactome pathway database was used for the bioinformatics analysis. A total of 15 studies were curated and 1717 common interactors were uncovered among all these studies. KEGG analysis, Enrichr analysis, STRING interaction analysis was performed on these interactors. Therefore, we have identified novel host pathways that can be targeted for host-directed therapy against influenza in our review.

## Introduction

Influenza viruses are negative-sense single-stranded RNA viruses from the *Orthomyxoviridae* family that cause respiratory diseases (MacLachlan and Dubovi, 2017). Of the 4 influenza virus types, A, B, C, and D, type A is the most prolific as it infects numerous hosts and is the main causative agent of the seasonal and pandemic influenza (Shen et al., 2015). Influenza viruses constantly evolve with antigenic shifts (reassortment of viral segments, resulting in dramatically different viruses) and drifts (small antigenic changes to increase immune evasion). Due to the viral adaptation and reassortment, highly virulent strains may appear and result in local epidemics or global pandemics, such as the 1918 H1N1 Spanish pandemic, 2005 H5N1 Bird flu, and the 2009 H1N1 Swine flu (Shen et al., 2015). Influenza’s genome, which is composed of eight segments of symmetrical helixes, encodes ten proteins. Those include the surface glycoproteins haemagglutinin (HA) and neuraminidase (NA), matrix protein (M1), matrix ion channel (M2), Nucleoprotein (NP), PA (polymerase acid subunit), polymerase basic subunit 1 (PB1), and polymerase basic subunit 2 (PB2), which form the RNA-dependent RNA polymerase complex, NS1 (non-structural protein 1) and NS2, non-structural protein 2 or nuclear export protein (NEP; Noda, 2012). Segments of some influenza A virus strains may encode a second or third polypeptide in alternative reading frames (McCauley et al., 2012). These functional proteins (Jagger et al., 2012), such as PB1-F2 and PA-X, are known to modulate the host response to the virus (Klemm et al., 2018). The influenza A virus can be further classified based on its HA and NA glycoproteins into different subtypes. There are 18 haemagglutinin and 11 neuraminidase subtypes known today (Tong et al., 2013). Only the H1, H2, H3, N1, and N2 subtypes have caused epidemics in humans (Bouvier and Palese, 2008).

### Current Prophylaxis and Treatment of Influenza

Vaccination is available for influenza and is the main form of prevention against influenza. Vaccine efficacy is around 60% if matched to the current circulating strains of the virus, but effectiveness can be as low as 10–20% if there is a mismatch between the vaccine and current strains of the virus (Eisenstein, 2019). The current method of developing influenza vaccines is lengthy, with the Centre for Disease Control (CDC) characterizing around 2,000 influenza viruses. These viruses are monitored for drifts and shifts and compared to viruses included in the current influenza vaccine. This provides an indication of the vaccine’s ability to produce an immune response against current circulating strains of influenza (CDC, 2019). There are three main kinds of vaccines: egg-based vaccines, attenuated vaccines, and recombinant vaccines. However, due to the constantly evolving nature of influenza, there are problems associated with vaccine mismatches (Paules et al., 2018) and poor immunogenicity of vaccines in the elderly (DiazGranados et al., 2014). Moreover, there is a lack of a universal influenza vaccine covering all strains and subtypes of influenza (Krammer et al., 2018).

Hence, there is a need for antiviral therapy for breakthrough infections and for infection with influenza strains not covered by vaccines. M2 ion channel inhibitors (e.g., amantadine and rimantadine) and NA inhibitors (e.g., oseltamivir and zanamivir) are the original two drug classes approved for influenza treatment (Krammer et al., 2018). Resistant virus strains have emerged and have rendered M2 inhibitors ineffective for clinical use while there is also increasing NA inhibitor resistance, such as the H274Y mutation, found in the 2009 H1N1 strain which conferred oseltamivir resistance (Arias et al., 2009; Shen et al., 2015). Recently, baloxavir (cap-dependent endonuclease inhibitor) was approved by the FDA in 2018 and worked by interfering influenza’s ability to multiply *via* inhibition of viral transcription (Hayden et al., 2018). There are a limited number of compounds under development or in trials (Davidson, 2018). Hence, there is an increasing focus to research and developing host-directed therapies given there is a lower drug resistance potential (Lou et al., 2014). We hypothesized that by combining various influenza interactome studies, there might be novel insights into viral–host interactors and processes that could be targeted for antiviral therapy. In this study, we identified novel host interactors of influenza *via* literature and database search. We further analyzed the data set by bioinformatics. This resulted in the identification of core cellular processes and druggable targets that could be further studied. This provides an overall landscape of conserved host processes targeted by various influenza strains for future drug development and better understanding the influenza life cycle.

## Materials and Methods

### Data Collection

In order to identify host interactions that are ubiquitous across the various influenza A strains, data was extracted from the Reactome database (Fabregat et al., 2018; Jassal et al., 2020) and Host Pathogen Interaction Database (HPIDB; Ammari et al., 2016) while a PubMed and Scopus search of primary literature that performed interaction studies. Papers were chosen if they had found specific interactions between a host protein and influenza proteins. Host interactions with viral complexes and novel accessory viral proteins, such as PA-X and PB1-F2, were excluded. This is because not all influenza A strains express these accessory proteins. A total of 15 papers were retrieved, and their methodology, as well as virus strain (s) studied, are listed Supplementary Table S1. Reactome is a free, online, curated, open-source pathway database contains the influenza infection pathway (REACT_6167.3), specifically NS1 Mediated Effects on Host Pathways (Homo sapiens). This pathway was last reviewed on 1 May 2007, thus does not contain any information from the studies utilized in this project. Despite its age, it was still included as a point of reference for subsequent analysis. (Fabregat et al., 2018; Jassal et al., 2020).

HPIDB was chosen as it contains a comprehensive set of host–virus interactions (Ammari et al., 2016). This includes experimentally derived HPI, predicted HPI *via* network analysis, and molecular interactions from other databases which include VirHostNet^1^ and UniProtKB (Consortium, 2012).^2^ Currently, in its third version, it has 69,787 unique protein interactions between 66 host and 668 pathogen species. In this project, all ten characterized influenza protein interactions (HA, NA, PA, PB1, PB2, NP, M1, M2, NS1, and NS2) from various influenza A strains with human proteins were extracted from the various studies and databases, compiled, and matched to reveal which are key interactors of influenza. The compiled data can be found in Supplementary Table S1.

### Data Set Analysis

**Figure 1:**
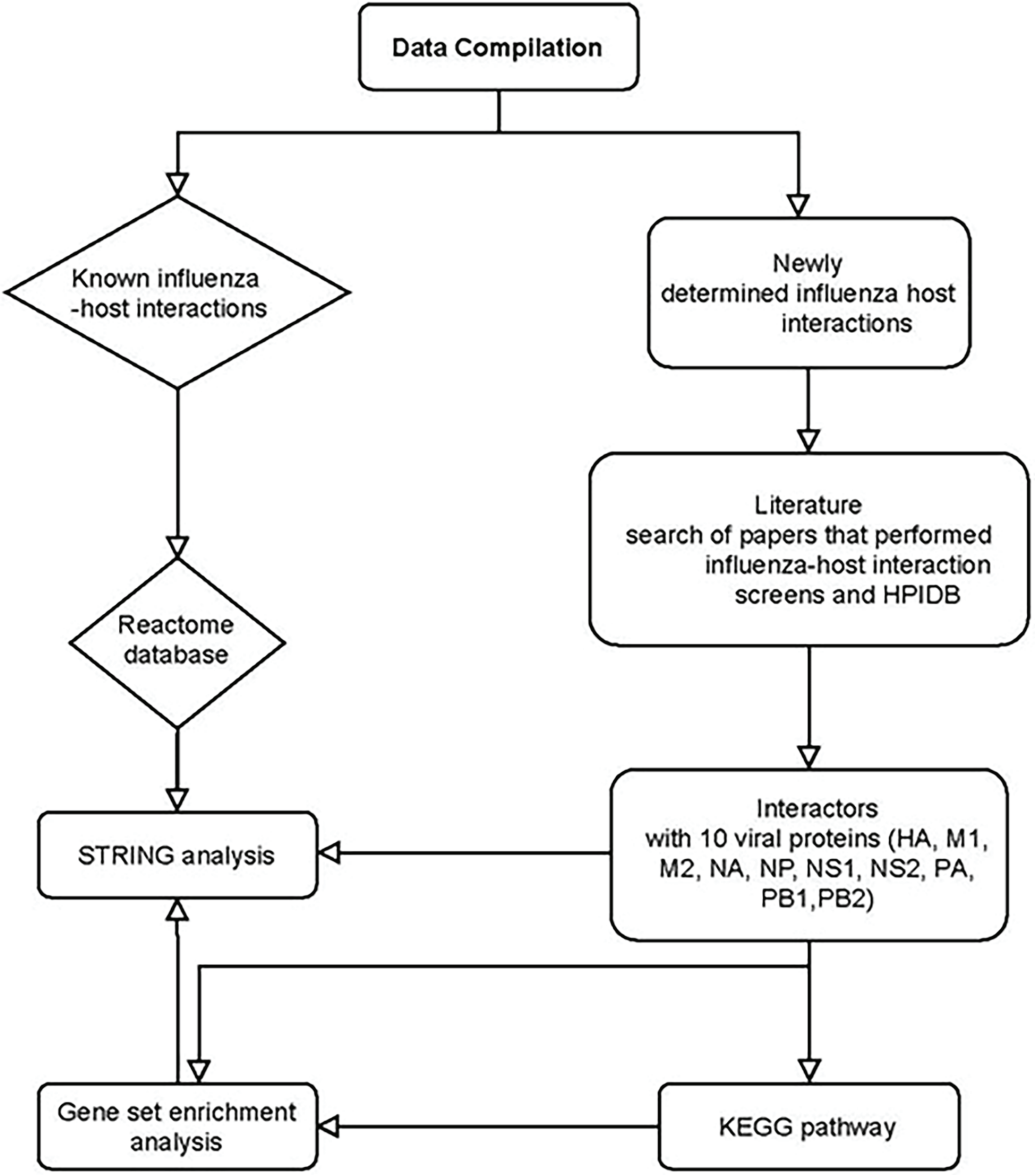
Bioinformatics analysis of influenza and host interactors. HPIDB: Human Pathogen Interactions Database. Host interactors of influenza were separated into known and newly determined interactions. PubMed search of Viral–host interactome studies (IP-MS, Y2H, computer homology) was conducted. Papers were filtered if they were in English and full data set was available for analysis. Compiled data set of interactors was analyzed using STRING, Gene set enrichment analysis, and KEGG pathway.

Using the filtered gene data set, we performed the following analysis. Enrichr (Chen et al., 2013; Kuleshov et al., 2016) was used for gene set enrichment analysis. Kyoto Encyclopedia of Genes and Genomes (KEGG)^3^ pathwayanalysis was conducted to identify genes at the biologically functional level (Kummer et al., 2014). For all enrichment analysis, a value of p cutoff of 0.05 was used as significant. Figure 1 shows the analysis workflow for the bioinformatics analysis.

## Results and Discussion

### Meta-Analysis of Influenza–Host Interactions

In order to better understand the landscape of influenza–host interactions, we performed an analysis of influenza host interactors. We compiled virus–host protein–protein interaction data from HPIDB, Reactome, and published interactome studies. The published studies and their methodology are described in Supplementary Table S1. This data set covered various strains of influenza A, such as H3N2, H1N1, and H7N9. This data set contained protein interactions from different experimental methods—Affinity purification-mass spectrometry (AP-MS), Yeast 2 hybrid (Y2H), RNA immunoprecipitation, and bioinformatics prediction of interactions. Given that single interactome studies may result in false positives, a host interactor was considered to be true if it appeared in at least 3 different studies and databases. Altogether, our review uncovered 1,717 host interactions among the ten viral proteins. Both KEGG and Enrichr functional analysis were performed for the interactors of each viral protein. Details of the specific host–viral interactions can be found from Supplementary Table S2.

### HA Interactors Are Mainly Involved in Protein Processing

HA is a trimer of identical subunits, each containing two polypeptides that result from proteolytic cleavage of a singular precursor (Skehel and Wiley, 2000). This cleavage is essential to activate membrane fusion potential and hence infectivity (Garten and Klenk, 1999; Steinhauer, 1999). A newly synthesized 70 kDa HA is cleaved into HA1 and HA2, which are linked by disulfide bonds. HA1 contains the sialic acid binding site. After binding, the virus is internalized into endosomes. Endosomal acidification triggers a marked and irreversible change in HA, which results in the dissociation of HA1 from the endosomal membrane and HA1 moving away from HA2. There is a loop-to-helix transition in HA2 which enables the fusion peptide at the N-terminus of HA2 to attach to the endosomal membrane. This promotes the fusion of the viral and endosomal membranes and this results in the vRNP release into the cytoplasm (Das et al., 2010).

HA binding to sialic acid receptor determines the species-specific infectivity of the influenza virus (Rogers and Paulson, 1983). Avian and equine viruses prefer α-2,3-galactose-linked sialic acid, human viruses prefer α-2,6-linked sialic acid and swine viruses appear to bind to both linkages of sialic acid (Rogers and D’Souza, 1989; Gambaryan et al., 1997; Ito et al., 1998).

**Figure 2:**
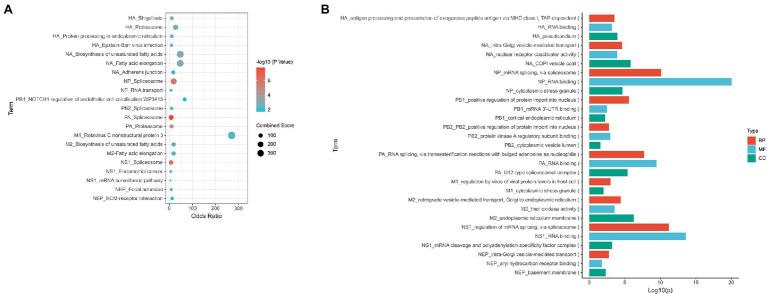
Summary of the main processes found to be represented for each viral protein. **(A)** Enriched KEGG pathway for interactors of each viral protein **(B)** GO analysis of interactors of each viral protein. Proteins were analyzed using Enrichr BP, MF, and CC represent Biological Process, Molecular Function, and Cellular Component groups of gene ontology (GO).

Based on our meta-analysis, 36 common host interactors were found across the various studies. The top KEGG pathway identified for HA interactors was the proteasomal pathway (i.e., PSMD6 and PSMD7), protein processing in endoplasmic reticulum (i.e., RPN1, CALR, and PDIA6), and adherens junction (i.e., ACTN1 and ACTN4; Figure 2A; Supplementary Figure S1A). The functional analysis of the interactors revealed that they were mainly involved in the immune pathway (i.e., PSMD6, PSMD7, ACTN4, ARF1, and ANXA2), protein processing (i.e., PSMD6, PSMD7, CALR), and post-translational modification (i.e., PSMD6, PSMD7, CALU, and PDIA6; Figure 2B; Supplementary Figure S1B). Given that HA is being transcribed and translated in the infected cell during the viral life cycle, this would point to the importance of the protein processing being key in influenza replication. Any drug targeting this process would affect the formation of new virions. This is supported by a previous study which showed HA being synthesized by ER-bound biosynthetic machinery and interacting with ER chaperone proteins calnexin and calreticulin (Hebert et al., 1997). Any drug targeting this process would affect the formation of new virions. Moreover, HA requires glycosylation for binding to sialic receptors (de Vries et al., 2010), while palmitoylation of HA is essential for the virus to form infectious virions (Chen et al., 2005). Therefore, this presents a key druggable target for a new therapy to target or prevent influenza infection. DAS181 has already been developed as a sialidase fusion protein to prevent the binding of haemagglutinin to sialic acid receptors. It has reached late-stage clinical trials (Koszalka et al., 2017).

An interesting observation was that immune-related processes were highly enriched from the gene set analysis. A previous study had reported that HA subunit 1 drove the IFN receptor chain IFNAR1 degradation, thus suppressing IFN-triggered JAK/STAT. The reduced JAK/STAT activation would lead to lower type I interferon production, resulting in decreased immune response and thereby increasing viral replication (Xia et al., 2016). This would confirm that HA is involved in regulating the host immune response as part of the influenza life cycle.

### NA Interactors Are Involved in Vesicle Transport

NA is a mushroom-shaped protein and is found as a tetramer of identical subunits, with the mushroom head suspended from the viral membrane on a thin, long stalk. Each subunit that forms the mushroom head is made up of a six-bladed propeller-like structure (Varghese et al., 1983).

During viral replication, NA removes sialic acid from cellular glycoproteins and glycolipids, as well as from both viral glycoproteins. This prevents newly assembled viruses from rebinding to the infected cell surface and with self-aggregate through HA-sialic acid interactions. New virions are then released from the cell to infect new cells and further the infection spread (Gamblin and Skehel, 2010). It is also thought that NA aids viral infectivity by breaking down the mucins in the respiratory tract secretions to allow the penetration of the virus to the respiratory epithelium and may play a role in viral entry into respiratory epithelial cells (Matrosovich et al., 2004).

A total of 36 NA interactors were found across the various studies. These proteins are involved in fatty acid metabolism (i.e., TECR and HACD3); focal adhesion tight and adherens junction (i.e., ACTN1 and ACTN4), and cell cycle (i.e., MCM7 and PRKDC) *via* KEGG analysis (Figure 2A; Supplementary Figure S2A). Functional analysis of NA interactors found that the most highly enriched processes were intra-Golgi vesicle transport and vesicle transport (i.e., COPB2, COPA, and COPG1; Figure 2B; Supplementary Figure S2B). Given that acetylation of α-tubulin occurs as part of the viral release (Husain and Harrod, 2011), this would suggest that NA is involved in this mechanism.

### NP, PB1, PB2, PA Interactors Are Involved in Spliceosome Activity

NP is a structural protein with no enzymatic activity but is the most abundant viral protein in infected cells (Hu et al., 2017). It is an important part of the vRNP complex and its functions include RNA packing (Eisfeld et al., 2015), nuclear trafficking (Amorim et al., 2013; Chutiwitoonchai and Aida, 2016), and vRNA transcription and replication (Eisfeld et al., 2015). A NP monomer has a molecular weight of 56 kDa that is able to bind to 24 bases of RNA (Hu et al., 2017). It is crescent-shaped with head, body, RNA binding, and tail domains (Cianci et al., 2013). The residues in the basic loop (residues 73–91) were found to be required for RNA binding (Ng et al., 2008). NP oligomerization occurs *via* a flexible tail-loop (residues 402–428) that can insert into the body domain of a neighboring NP monomer (Ye et al., 2006). This tail insertion is facilitated by R419 and E339 which forms a critical salt bridge for stabilization (Cianci et al., 2013). NP also directly interacts with PB1 and PB2 subunits of the viral polymerase (Biswas et al., 1998; Fodor, 2013; Eisfeld et al., 2015; Davis et al., 2017). The C-terminus of NP (aa 340 to 498) contains a PB2 binding site and a sequence that regulates the NP-PB2 interaction. In addition to its role in the vRNP, NP has been found to induce apoptosis in host cells (Tripathi et al., 2013; Nailwal et al., 2015) and inhibit PKR activation *via* Hsp40 (Sharma et al., 2011).

The vRNP polymerase complex is a heterotrimer formed together by PB1 with PB2 and PA in the viral polymerase (Eisfeld et al., 2015). PB1 itself has the polymerase activity and is enclosed by the PA linker on one side (Ma et al., 2017) and the N-terminal domain of PB2 at the other side (Stevaert and Naesens, 2016). PA contains the endonuclease domain while PB2 has the cap-binding domain (te Velthuis and Fodor, 2016). The LLFL motif in PB1 N-terminus (residue 7–10) interacts with the PA C-terminus hydrophobic core (F411, M595, L666, W706, F710, V636, and L640; Massari et al., 2016). Based on a crystal structure, the C-terminus of PB1 (residues 678–757) was found to complex with the N-terminus of PB2 (residues 1–37; Sugiyama et al., 2009).

Among the three subunits of the vRNP polymerase complex, PB1 had the least interactome studies and the least number of interactors. The most identified interactor for PB1 was PP6R3. Among all the studies, PA had 316 interactors while NP had 51 interactors and PB2 had 45 interactors. The interactors of NP, PB2, and PA are mostly involved in spliceosome based on the KEGG pathway (Figure 2A; Supplementary Figures S3A–C). The common process for all the interactors of NP, PB2, and PB1 is the transportation of proteins into the nucleus (i.e., NCBP1, SRSF1, PHAX, U2AF1, SRRM1, BAG3, UBR5, and IPO5; Figure 2B; Supplementary Figures S3D–G). This is expected as the vRNP is required to enter the nucleus for viral replication. In addition, spliceosome pathway is a common process for the interactors of all the vRNP components (i.e., SF3B4, DDX5, SF3B2, SF3B3, SF3B6, SRSF1, U2AF1, CHERP, TRA2B, DHX15, SRSF3, SRSF6, SRSF7, SF3B1, RBMX, DDX5, FXR2, NCBP1, PCBP1, and SNRPA). These interactors are also involved in general RNA processing (Supplementary Figures S3D–G). This may explain the spliceosome being identified with these host interactors, since the spliceosome is part of the RNA processing pathway (Licatalosi and Darnell, 2010; Wilkinson et al., 2020). This is a unique observation as NS1 has traditionally been the viral protein associated with spliceosome inhibition due to its binding to CPSF4 (Twu et al., 2006; Ramos et al., 2013). It was previously reported that the vRNP complex is required to stabilize the NS1-CPSF30 complex, specifically NP and PA (Kuo and Krug, 2009). However, the role of the viral polymerase complex alone in spliceosome regulation has yet to be studied.

The proteasome pathway was a specific pathway identified for PA interactors (i.e., PSMD6, PSMD7, PSMD4, PSMD2, PSMD3, and PSMD1). This would correlate to other studies which has found that treatment with proteasome inhibitors resulted in an antiviral state in cells (Dudek et al., 2010; Haasbach et al., 2011). It was also reported that treatment with the clinical approved proteasomal inhibitor PS-341 resulted in degradation of IκB and the activation of NF-κB and JNK/AP-1 pathway (Dudek et al., 2010). Hence, this suggests that the proteasomal pathway may be present a novel method of targeting influenza.

An interesting finding was that Annexin A2 (ANXA2) was identified as an interactor of PA across 3 papers (Bradel-Tretheway et al., 2011; Watanabe et al., 2014; Heaton et al., 2016) and HPIDB. Previously, it was reported that ANXA2 binds to highly pathogenic H5N1 influenza NS1 to enhance viral replication. Moreover, ANXA2 is incorporated into IAV particles to enhance viral replication, *via* the conversion of plasminogen to plasmin (LeBouder et al., 2008).

### M2 Interactors Are Involved in Fatty Acid Metabolism

The M gene encodes for both M1 and M2 proteins (Lamb et al., 1981). M1 protein consists of 252 amino acids, with 2 domains (N-terminal domain from amino acid 1 to 164 and the C-terminal domain from amino acid 165 to 252) linked by a protease-sensitive loop (Ito et al., 1991). It forms the matrix layer by oligomerizing directly below the lipid envelope and binds the viral ribonucleoproteins. It has the important function of stabilizing the whole envelope structure of a fully formed virion (Harris et al., 2001; Calder et al., 2010; Schaap et al., 2012; Adachi et al., 2017). M1 contacts with both viral RNA and NP, promoting the vRNP complex formation and cause the RNP dissociation from the nuclear matrix (Wakefield and Brownlee, 1989; Elster et al., 1994, 1997; Nasser et al., 1996; Chaimayo et al., 2017). M1 plays an important role in assembly by recruiting viral components to the assembly and an essential role in budding, such as viral particle formation (Gómez-Puertas et al., 2000; Latham and Galarza, 2001). M1 had the least interactome studies and only three common interactors were found: EZRI, HSP7C, and STAU1. Based on these three interactors, the interactors were found to be positive regulators of virus replication (Figure 2; Supplementary Figures S4A,B).

The M2 protein comprises of 97 amino acids with three domains: extracellular (24 amino acids), transmembrane domain (19 amino acids), and cytoplasmic domain (54 amino acids). It is a membrane protein which is inserted into the viral envelope and projects from the surface of the virus as tetramers (Lamb et al., 1985; Holsinger and Alams, 1991). The M2 protein is a proton channel and is required in the acidification of the viral particle upon endocytosis (Lamb et al., 1985) and prior to membrane fusion to enable the release of vRNPs into the cytosol (Helenius, 1992). It is also required to prevent the Golgi lumen pH from becoming too acidic so that the nascent HA do not undergo premature conformational arrangement while being transported to the plasma membrane (Sugrue and Hay, 1991).

Eighty-nine host proteins were found to interact with M2 across the various interactome studies. The most common interactors were 4F2, AFG32, ECHB, SPTC1, and TMX3. KEGG analysis revealed that these proteins were mainly involved in fatty acid metabolism (i.e., TECR and HACD3) and DNA replication (i.e., RFC3 and MCM7; Figure 2A; Supplementary Figure S4C). Functional enrichment analysis showed that these proteins were involved in vesicle transport (i.e., COPB2, COPA, ZW10, GBF1, and COPG1; Figure 2B; Supplementary Figure S4D). Given that ubiquitination of M2 is required for viral packaging and release (Su et al., 2018), this would confirm the importance of the vesicle pathway for influenza *via* M2.

An interesting observation was the involvement of M2 interactors in fatty acid metabolism. Fatty acid oxidation was found to be reduced in influenza-infected mice (Ohno et al., 2020), while supplying palmitic acid increased influenza replication (Limsuwat et al., 2020). Influenza replication could be reduced by a fatty acid import inhibitor (Limsuwat et al., 2020). Hence, this would suggest that M2 may be involved in the fatty acid metabolism dysregulation caused by influenza infection. In addition, a recent study reported that M2 clustering which enables membrane scission is mediated by cholesterol (Elkins et al., 2017). Given that cholesterol is involved in viral membrane fusion, viral genome release, and viral budding, this may explain the efficacy of cholesterol-lowering drugs, gemfibrozil, and lovastatin in reducing the stability and infectivity of progeny virus (Bajimaya et al., 2017). It was also demonstrated that overexpression of Annexin A6 as well as the addition of U18666A, a hydrophobic polyamine, was able to reduce cholesterol levels in the plasma membrane and inhibit viral replication (Musiol et al., 2013).

### NS1 Interacting Partners Are Involved in Spliceosome and Autophagy

NS1 is not part of the virion structural component, but it is expressed at high levels in infected cells (Hale et al., 2008). It is composed of 231–237 amino acids, depending on the strain, and has a molecular mass of around 26 kDa (Hale et al., 2008). It has two distinct functional domains: an N-terminal RNA binding domain (amino acids 1–73) and a C-terminal effector domain (amino acids 86-231/237), which mediates binding with host cell proteins (Wang et al., 2002; Kochs et al., 2007; Hale et al., 2008; Das et al., 2010). NS1 was reported to have multiple functions that contribute to viral replication and virulence (Kochs et al., 2007; Hale et al., 2008; Fournier et al., 2014). These include: (i) temporarily regulating viral RNA synthesis (Hale et al., 2008; Ayllon and García-Sastre, 2015); (ii) viral mRNA splicing control (Hale et al., 2008, Ayllon and García-Sastre, 2015); (iii) enhancing viral mRNA translation *via* PKR inhibition (Li et al., 2006); (iv) regulating the creation of the virus particle structure (Hale et al., 2008; Pereira et al., 2017); (v) suppressing the host immune or apoptotic responses (Ehrhardt et al., 2007; Kochs et al., 2007; Iwai et al., 2010; Jia et al., 2010; Mata et al., 2011; Gao et al., 2012; Anastasina et al., 2016); (vi) activating phosphoinositide 3-kinase (Ehrhardt et al., 2007; Gaur et al., 2011; Ayllon and García-Sastre, 2015); and (vii) involvement in strain-dependent pathogenesis (Hale et al., 2008). NS1 exists as a homodimer. The RNA binding domain binds to RNA and the binding is dependent on R38 and other charged residues, such as R35 and K41 (Lalime and Pekosz, 2014; Ayllon and García-Sastre, 2015). In addition, the effector domain of NS1 has been found to bind to CPSF30, which results in reduced IFN-β mRNA production. The key amino acid residue for the CPSF30 interaction is W187 (Engel, 2013). Moreover, multiple mutations in NS1 have been found to increase virulence (Ozawa et al., 2011; DeDiego et al., 2016) and viral pathogenicity (Ehrhardt et al., 2007; Engel, 2013; Nogales et al., 2017).

NS1 had the most interactome studies among all the viral proteins. A total of 252 interactors were found among the interactome studies. The most common interactor found was STAU1 found in nine studies, followed by PRKRA. Based on the KEGG pathway, these interactors are involved in spliceosome (i.e., SF3B2, SNW1, FUS, NCBP2, PCBP1, TRA2B, TRA2A, DHX15, SRSF3, SRSF6, SRSF7, and RBMX) and autophagy (i.e., IRS1, BAD, IRS4, RAF1, and TANK; Figure 2A; Supplementary Figure S5A). Enrichr analysis also revealed that these interacting partners are involved in mRNA splicing (i.e., DDX17, PRDX6, QKI, FXR1, PTBP1, FXR2, SNW1, SON, TRA2B, TRA2A, SRSF3, SRSF6, SRSF7, and RBMX) and RNA processing (i.e., CPSF4, SF3B2, CHTOP, RBM14, CPSF1, FUS, NCBP2, CPSF2, PTBP1, SNW1, SON, PCBP1, TRA2B, TRA2A, DHX15, SRSF3, RALY, SRSF6, SRSF7, and RBMX; Figure 2B; Supplementary Figure S5B). NS1 is a well-known interactor of the spliceosome pathway, given its interactions with CPSF4 and NS1-BP (Wolff et al., 1998; Thompson et al., 2018; Zhang et al., 2018). It has been previously reported that NS1 interacts with hnRNP-F to modulate host mRNA processing (Lee et al., 2010). In addition, NS1 is required for unspliced M1 nuclear export (Pereira et al., 2017). NS1 is also a well-known inducer of autophagy (Zhirnov and Klenk, 2013; Zhang et al., 2019). It was previously reported that NS1 induced autophagy *via* its interaction with JNK (Zhang et al., 2019).

### NS2 Interactors Are Mainly Involved in Focal Adhesion and ECM–Receptor Interaction

NEP or non-structural protein 2 (NS2) is a structural protein and is associated with M1 (Yasuda et al., 1993). NEP mediates vRNP nuclear export into the cytoplasm *via* an export signal (O’Neill et al., 1998) through XPO1 interaction (Neumann et al., 2000). NEP has also been found to interact with nucleoporins and is suggested to act as an adaptor between vRNPs and the nuclear pore complex (O’Neill et al., 1998). It has been proposed that NEP is involved in the transcription and replication of the influenza virus (Robb et al., 2009).

Forty host proteins were found to be NS2 interactors across the different studies. These proteins are involved in focal adhesion and ECM–receptor interaction (i.e., LAMC3, ZYX, and LAMB1), and ubiquitin-mediated proteolysis (i.e., PIAS3 and SKP1) *via* KEGG analysis (Figure 2A; Supplementary Figure S6A). Functional enrichment analysis showed that these proteins are involved in microtubule reorganization (i.e., DCTN2, CENPH, and ZWINT) and ER-Golgi transport (i.e., COG8 and COG6; Figure 2; Supplementary Figure S6B).

### Influenza Interacting Partners Are Involved in the Spliceosome, Focal Adhesions, and Protein Processing in the ER

A global analysis of the protein interactors revealed that most of these interactors are involved in the spliceosome, followed by focal adhesion and protein processing in the ER *via* KEGG analysis. Given the large proportion of NS1 interactome studies, this may have resulted in spliceosome being the top process. However, it was interesting to note that focal adhesion and protein processing in the ER was one of the top processes revealed for influenza interactors.

A previous study revealed that actin reorganization is required for influenza assembly and budding *via* cofilin-1 phosphorylation (Liu et al., 2014). Focal adhesion kinase (FAK) is a non-receptor tyrosine kinase (non-PTK) and part of focal adhesions that tether actin cytoskeleton to the extracellular matrix. It was previously shown that FAK is involved in phosphatidylinositol-3-kinase (PI3K) activation and reorganization of cytoskeleton for endosomal trafficking during IAV entry (Elbahesh et al., 2014). In addition, FAK was found to positively regulate IAV replication and polymerase activity of different IAV strains (Elbahesh et al., 2016). FAK-dependent regulation of innate immune responses was observed during severe IAV infection in mice (Bergmann and Elbahesh, 2019). Given that HA, NA, and NS2 have interactors involved in focal adhesions or ECM, this may be a potential target for novel influenza therapy.

**Figure 3:**
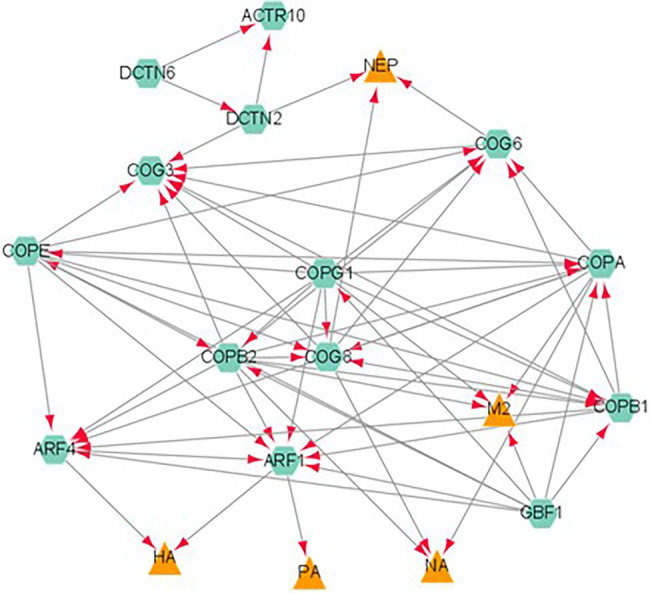
A network analysis of influenza proteins and host proteins involved in ER protein processing. Proteins classified as belonging to the ER protein processing pathway were analyzed by STRING database. The viral proteins are represented by orange triangles and host interactors are represented by green hexagons. Each interaction is represented by edges connecting the two nodes with the arrow reflecting a positive association between two proteins.

In addition, a novel finding was the involvement of these interactors in protein processing in the ER. Figure 3 describes the interaction of the influenza proteins and host proteins involved in ER protein processing. It was previously reported that influenza modulates vesicle trafficking *via* disruption of the Golgi complex (Yadav et al., 2016). In addition, brefeldin A, an ER to Golgi transport inhibitor, was found to affect the intracellular distribution of HA (Russ et al., 1991; Ciampor et al., 1997) and induce apoptosis in influenza-infected cells (Saito et al., 1996). Monensin, a Golgi complex disruptor, was found to reduce viral budding and HA localization to the cell membrane (Edwardson, 1984).

Given the role of influenza proteins, NS1, NP, PA, and PB1 in spliceosome activity, targeting this pathway may be a potential target for influenza. Cdc2-like kinase 1 (CLK1) is a kinase that regulates alternative splicing of pre-mRNA (Bullock et al., 2009). CLK1 inhibition by TG003 or CLK1 knockdown was shown to decrease M2 mRNA generation and downstream M2 protein expression, thus reducing IAV propagation (Karlas et al., 2010).

A total of 190 host proteins had more than 1 viral protein interaction. 4F2 was the most promiscuous host protein with 6 viral protein interactions. ADT3, ANXA2, PSD11, PSD12, PSD13, and TCPE had 5 viral protein interactions each. Given that these proteins have interactions with multiple viral proteins, this may indicate that these proteins are key for multiple aspects of viral replication and life cycle and, hence, should be further studied for novel host-directed therapy.

## Conclusions and Future work

Identifying novel host interactors in influenza is key in understanding the viral life cycle as well as for the development of novel therapies against the virus. Our review of influenza interactors has comprehensively compiled influenza interactome studies together with host–virus interaction databases. While other studies have done interactome studies on influenza proteins, no study has done a comprehensive review on influenza interactors. Using interactome studies to derive our data set also enables a more direct virus–host interaction compared to RNAi studies and would include essential genes which may lead to cell death in RNAi studies, thus reducing false negatives (Watanabe et al., 2010). While Watanabe et al. (2010) and Tripathi et al. (2015) both did meta-analysis of RNAi studies, no other study has done a meta-analysis of interactome studies for influenza. Our study has revealed novel influenza interactors which can be potentially targeted for novel therapies against influenza. It also filters out possible false positives that may be derived from a single interactome study. Given that we used a benchmark of at least three separate studies to filter out true positive, this would reflect the sensitivity of our study to detect conserved host interactors of influenza. Moreover, given that our study included various strains of influenza A, this would increase the likelihood that these pathways are globally used by all influenza A strains for their life cycle. Hence, these pathways can be further studied as potential universal therapy against influenza.

Based on our bioinformatics analysis of influenza proteins, we observed that protein transport to the ER is one of the top biological processes exploited by influenza (Figure 2). This supports the previous study by Heaton et al. (2016), where they identified Sec61 knockdown was found to reduce influenza replication. This suggests that the protein transport to the ER is one of the key processes that influenza exploits for its life cycle. In addition, ER transport is a key process in the innate immunity. A member of the COPII complex, Sec13, was previously identified in a CRIPSR knockout screen to reduce influenza replication (Li et al., 2020). This would support the important role of ER processing for influenza. Hence, more studies into how it can be targeted for influenza treatment should be undertaken. Several pro-inflammatory cytokines, such as IL6 and IFN-β, are secreted (Murray and Stow, 2014) *via* the ER-Golgi pathway. Influenza’s control over this pathway would enable it to replicate without detection from immune cells and this would reflect the importance of the protein transport system. Moreover, influenza’s ability to overstimulate the immune response *via* cytokine storm has been shown to be correlated to virus virulence (Kido, 2015; Li et al., 2018; Short et al., 2018). This correlates with other RNAi studies performed in influenza which reflect the importance of the ER to Golgi transport pathway in viral replication (Tripathi et al., 2015). Given the importance of post-translational modification of influenza proteins in viral replication and host response, as discussed by Hu et al. (2020), this would be a potential target for influenza treatment, especially in the context of severe influenza. This finding can be extrapolated to other viruses as discussed in the review (Ravindran et al., 2016). Both enveloped and non-enveloped viruses were described to hijack the ER for replication. HIV utilizes the ER to synthesize its envelope glycoprotein (Checkley et al., 2011). Antiviral therapeutics that impair ER-resident glycan trimming enzymes α-glucosidases I and II have been shown inhibit viral infection by DNA and RNA viruses (Chang et al., 2013). Moreover, an inhibitor against HSP70, a cytosolic chaperone that controls ER-associated degradation, has been shown to inhibit flavivirus infection (Taguwa et al., 2015).

Future work would be to validate the targets identified in this review *via in vitro* and *in vivo* models. However, this review has reflected the key processes that can be potentially targeted for host-directed therapy against influenza. Given the key role these processes play in influenza as well as normal host cell maintenance, it would be important to find key differences between normal cellular maintenance and viral infection. This would enable specific targeting of influenza-driven pathways without killing the host. Another aspect that would need to be studied is how these processes contribute to severe influenza, which is still currently unknown. Therefore, a more detailed analysis of IAV–host interactions would provide clues for therapeutic targeting and molecular mechanisms of viral replication.

## Author Contributions

SC, JC, DE, and LL designed the experiments. SC performed the experiments. SC, JC, and LL analyzed the data. SC wrote the manuscript. DE and LL did the final editing of the manuscript. All authors contributed to the article and approved the submitted version.

## Funding

LL and DE are supported by the Singapore National Research Foundation under its HUJ-NUS partnership program at the Campus for Research Excellence and Technology Enterprise (CREATE). SC is funded by a PHD scholarship from NUS and the Hebrew University. This study was funded by a grant from the National Research Foundation of Singapore (SHARE MMID-2).

## Conflict of Interest

The authors declare that the research was conducted in the absence of any commercial or financial relationships that could be construed as a potential conflict of interest.

## Publisher’s Note

All claims expressed in this article are solely those of the authors and do not necessarily represent those of their affiliated organizations, or those of the publisher, the editors and the reviewers. Any product that may be evaluated in this article, or claim that may be made by its manufacturer, is not guaranteed or endorsed by the publisher.

## Footnotes

^1^ http://virhostnet.prabi.fr

^2^ https://www.uniprot.org/

^3^ http://www.genome.jp/kegg/pathway.html

## References

[ref1] Adachi A. Koizumi M. Ohsumi Y. (2017). Autophagy induction under carbon starvation conditions is negatively regulated by carbon catabolite repression. J. Biol. Chem. 292, 19905–19918. doi: 10.1074/jbc.M117.817510, PMID: 29042435 PMC5712628

[ref2] Ammari M. G. Gresham C. R. Mccarthy F. M. Nanduri B. (2016). HPIDB 2.0: a curated database for host–pathogen interactions. Database 2016:baw103. doi: 10.1093/database/baw103, PMID: 27374121 PMC4930832

[ref3] Amorim M. J. Kao R. Y. Digard P. (2013). Nucleozin targets cytoplasmic trafficking of viral ribonucleoprotein-Rab11 complexes in influenza A virus infection. J. Virol. 87, 4694–4703. doi: 10.1128/JVI.03123-12, PMID: 23408618 PMC3624347

[ref4] Anastasina M. Le May N. Bugai A. Fu Y. Söderholm S. Gaelings L.. (2016). Influenza virus NS1 protein binds cellular DNA to block transcription of antiviral genes. Biochim. Biophys. Acta 1859, 1440–1448. doi: 10.1016/j.bbagrm.2016.09.005, PMID: 27664935

[ref5] Arias C. F. Escalera-Zamudio M. Soto-Del Rio Mde L. Cobian-Guemes A. G. Isa P. Lopez S. (2009). Molecular anatomy of 2009 influenza virus A (H1N1). Arch. Med. Res. 40, 643–654. doi: 10.1016/j.arcmed.2009.10.007, PMID: 20304251

[ref6] Ayllon J. García-Sastre A. (2015). “The NS1 protein: a multitasking virulence factor,” in Influenza Pathogenesis and Control – Volume II. eds. Oldstone M. B. A. Compans R. W. (Cham: Springer International Publishing). 10.1007/82_2014_400 25007846

[ref7] Bajimaya S. Frankl T. Hayashi T. Takimoto T. (2017). Cholesterol is required for stability and infectivity of influenza A and respiratory syncytial viruses. Virology 510, 234–241. doi: 10.1016/j.virol.2017.07.024, PMID: 28750327 PMC5571833

[ref8] Bergmann S. Elbahesh H. (2019). Targeting the proviral host kinase, FAK, limits influenza a virus pathogenesis and NFkB-regulated pro-inflammatory responses. Virology 534, 54–63. doi: 10.1016/j.virol.2019.05.020, PMID: 31176924

[ref9] Biswas S. K. Boutz P. L. Nayak D. P. (1998). Influenza virus nucleoprotein interacts with influenza virus polymerase proteins. J. Virol. 72, 5493–5501. doi: 10.1128/JVI.72.7.5493-5501.1998, PMID: 9621005 PMC110190

[ref10] Bouvier N. M. Palese P. (2008). The biology of influenza viruses. Vaccine 26(Suppl 4), D49–D53. doi: 10.1016/j.vaccine.2008.07.039 19230160 PMC3074182

[ref11] Bradel-Tretheway B. G. Mattiacio J. L. Krasnoselsky A. Stevenson C. Purdy D. Dewhurst S.. (2011). Comprehensive proteomic analysis of influenza virus polymerase complex reveals a novel association with mitochondrial proteins and RNA polymerase accessory factors. J. Virol. 85, 8569–8581. doi: 10.1128/JVI.00496-11, PMID: 21715506 PMC3165779

[ref12] Bullock A. N. Das S. Debreczeni J. É. Rellos P. Fedorov O. Niesen F. H.. (2009). Kinase domain insertions define distinct roles of CLK kinases in SR protein phosphorylation. Structure 17, 352–362. doi: 10.1016/j.str.2008.12.023, PMID: 19278650 PMC2667211

[ref13] Calder L. J. Wasilewski S. Berriman J. A. Rosenthal P. B. (2010). Structural organization of a filamentous influenza A virus. Proc. Natl. Acad. Sci. U. S. Am. 107, 10685–10690. doi: 10.1073/pnas.1002123107, PMID: 20498070 PMC2890793

[ref14] CDC (2019). Seasonal Influenza (Flu): Antigenic Characterization [Online]. Available at: https://www.cdc.gov/flu/about/professionals/antigenic.htm (Accessed 30 August, 2021).

[ref15] Chaimayo C. Hayashi T. Underwood A. Hodges E. Takimoto T. (2017). Selective incorporation of vRNP into influenza A virions determined by its specific interaction with M1 protein. Virology 505, 23–32. doi: 10.1016/j.virol.2017.02.008, PMID: 28219018 PMC5366082

[ref16] Chang J. Block T. M. Guo J. T. (2013). Antiviral therapies targeting host ER alpha-glucosidases: current status and future directions. Antiviral Res 99, 251–260. doi: 10.1016/j.antiviral.2013.06.011, PMID: 23816430 PMC7114303

[ref17] Checkley M. A. Luttge B. G. Freed E. O. (2011). HIV-1 envelope glycoprotein biosynthesis, trafficking, and incorporation. J. Mol. Biol. 410, 582–608. doi: 10.1016/j.jmb.2011.04.042, PMID: 21762802 PMC3139147

[ref18] Chen B. J. Takeda M. Lamb R. A. (2005). Influenza virus hemagglutinin (H3 subtype) requires palmitoylation of its cytoplasmic tail for assembly: M1 proteins of two subtypes differ in their ability to support assembly. J. Virol. 79, 13673–13684. doi: 10.1128/JVI.79.21.13673-13684.2005, PMID: 16227287 PMC1262586

[ref19] Chen E. Y. Tan C. M. Kou Y. Duan Q. Wang Z. Meirelles G. V.. (2013). Enrichr: interactive and collaborative HTML5 gene list enrichment analysis tool. BMC Bioinf. 14:128. doi: 10.1186/1471-2105-14-128, PMID: 23586463 PMC3637064

[ref20] Chutiwitoonchai N. Aida Y. (2016). NXT1, a novel influenza A NP binding protein, promotes the nuclear export of NP via a CRM1-dependent pathway. Viruses 8:209. doi: 10.3390/v8080209, PMID: 27483302 PMC4997571

[ref21] Ciampor F. Závodská E. Cmarko D. Cmarková J. Varecková E. (1997). Effects of brefeldin A on the expression and transport of influenza A virus haemagglutinin, M1 and M2 proteins within the cell. Acta Virol 41, 83–91. 9219638

[ref22] Cianci C. Gerritz S. W. Deminie C. Krystal M. (2013). Influenza nucleoprotein: promising target for antiviral chemotherapy. Antiviral Chem. Chemother. 23, 77–91. doi: 10.3851/IMP2235, PMID: 22837443

[ref23] Consortium U. (2012). Update on activities at the universal protein resource (UniProt) in 2013. Nucleic Acids Res. 41, D43–D47. doi: 10.1093/nar/gks1068, PMID: 23161681 PMC3531094

[ref24] Das K. Aramini J. M. Ma L. C. Krug R. M. Arnold E. (2010). Structures of influenza A proteins and insights into antiviral drug targets. Nat Struct Mol Biol 17, 530–538. doi: 10.1038/nsmb.1779, PMID: 20383144 PMC2957899

[ref25] Davidson S. (2018). Treating influenza infection, from now and into the future. Front. Immunol. 9:1946. doi: 10.3389/fimmu.2018.01946, PMID: 30250466 PMC6139312

[ref26] Davis A. M. Ramirez J. Newcomb L. L. (2017). Identification of influenza A nucleoprotein body domain residues essential for viral RNA expression expose antiviral target. Virol. J. 14, 22. doi: 10.1186/s12985-017-0694-8, PMID: 28173821 PMC5294902

[ref27] De Vries R. P. De Vries E. Bosch B. J. De Groot R. J. Rottier P. J. De Haan C. A. (2010). The influenza A virus hemagglutinin glycosylation state affects receptor-binding specificity. Virology 403, 17–25. doi: 10.1016/j.virol.2010.03.047, PMID: 20441997

[ref28] Dediego M. L. Nogales A. Lambert-Emo K. Martinez-Sobrido L. Topham D. J. (2016). NS1 protein mutation I64T affects interferon responses and virulence of circulating H3N2 human influenza A viruses. J. Virol. 90, 9693–9711. doi: 10.1128/JVI.01039-16, PMID: 27535054 PMC5068522

[ref29] Diazgranados C. A. Dunning A. J. Kimmel M. Kirby D. Treanor J. Collins A.. (2014). Efficacy of high-dose versus standard-dose influenza vaccine in older adults. New Eng. J. Med. 371, 635–645. doi: 10.1056/NEJMoa1315727, PMID: 25119609

[ref30] Dudek S. E. Luig C. Pauli E.-K. Schubert U. Ludwig S. (2010). The clinically approved proteasome inhibitor PS-341 efficiently blocks influenza A virus and vesicular stomatitis virus propagation by establishing an antiviral state. J. Virol. 84, 9439–9451. doi: 10.1128/JVI.00533-10, PMID: 20592098 PMC2937650

[ref31] Edwardson J. M. (1984). Effects of monensin on the processing and intracellular transport of influenza virus haemagglutinin in infected MDCK cells. J. Cell Sci. 65, 209–221. doi: 10.1242/jcs.65.1.209, PMID: 6425306

[ref32] Ehrhardt C. Wolff T. Pleschka S. Planz O. Beermann W. Bode J. G.. (2007). Influenza A virus NS1 protein activates the PI3K/Akt pathway to mediate antiapoptotic signaling responses. J. Virol. 81, 3058–3067. doi: 10.1128/JVI.02082-06, PMID: 17229704 PMC1866065

[ref33] Eisenstein M. (2019). Towards a universal flu vaccine. Nature 573, S50–S52. doi: 10.1038/d41586-019-02751-w, PMID: 31534251

[ref34] Eisfeld A. J. Neumann G. Kawaoka Y. (2015). At the centre: influenza A virus ribonucleoproteins. Nat. Rev. Microbiol. 13, 28–41. doi: 10.1038/nrmicro3367, PMID: 25417656 PMC5619696

[ref35] Elbahesh H. Bergmann S. Russell C. J. (2016). Focal adhesion kinase (FAK) regulates polymerase activity of multiple influenza A virus subtypes. Virology 499, 369–374. doi: 10.1016/j.virol.2016.10.002, PMID: 27743963

[ref36] Elbahesh H. Cline T. Baranovich T. Govorkova E. A. Schultz-Cherry S. Russell C. J. (2014). Novel roles of focal adhesion kinase in cytoplasmic entry and replication of influenza A viruses. J. Virol. 88, 6714–6728. doi: 10.1128/JVI.00530-14, PMID: 24696469 PMC4054363

[ref37] Elkins M. R. Williams J. K. Gelenter M. D. Dai P. Kwon B. Sergeyev I. V.. (2017). Cholesterol-binding site of the influenza M2 protein in lipid bilayers from solid-state NMR. Proc. Nat. Acad. Sci. 114, 12946–12951. doi: 10.1073/pnas.1715127114, PMID: 29158386 PMC5724280

[ref38] Elster C. Fourest E. Baudin F. Larsen K. Cusack S. Ruigrok R. W. H. (1994). A small percentage of influenza virus M1 protein contains zinc but zinc does not influence in vitro M1-RNA interaction. J. Gen. Virol. 75, 37–42. doi: 10.1099/0022-1317-75-1-37 8113738

[ref39] Elster C. Larsen K. Gagnon J. Ruigrok R. W. Baudin F. (1997). Influenza virus M1 protein binds to RNA through its nuclear localization signal. J. Gen. Virol. 78, 1589–1596. doi: 10.1099/0022-1317-78-7-1589, PMID: 9225034

[ref40] Engel D. A. (2013). The influenza virus NS1 protein as a therapeutic target. Antiviral Res. 99, 409–416. doi: 10.1016/j.antiviral.2013.06.005, PMID: 23796981 PMC4373342

[ref41] Fabregat A. Sidiropoulos K. Viteri G. Marin-Garcia P. Ping P. Stein L.. (2018). Reactome diagram viewer: data structures and strategies to boost performance. Bioinformatics 34, 1208–1214. doi: 10.1093/bioinformatics/btx752, PMID: 29186351 PMC6030826

[ref42] Fodor E. (2013). The RNA polymerase of influenza a virus: mechanisms of viral transcription and replication. Acta Virol. 57, 113–122. doi: 10.4149/av_2013_02_113, PMID: 23600869

[ref43] Fournier G. Chiang C. Munier S. Tomoiu A. Demeret C. Vidalain P. O.. (2014). Recruitment of RED-SMU1 complex by influenza A virus RNA polymerase to control viral mRNA splicing. PLoS Pathog. 10:e1004164. doi: 10.1371/journal.ppat.1004164, PMID: 24945353 PMC4055741

[ref44] Gambaryan A. S. Tuzikov A. B. Piskarev V. E. Yamnikova S. S. Lvov D. K. Robertson J. S.. (1997). Specification of receptor-binding phenotypes of influenza virus isolates from different hosts using synthetic sialylglycopolymers: non-egg-adapted human H1 and H3 influenza A and influenza B viruses share a common high binding affinity for 6′-sialyl(N-acetyllactosamine). Virology 232, 345–350. doi: 10.1006/viro.1997.8572, PMID: 9191848

[ref45] Gamblin S. J. Skehel J. J. (2010). Influenza hemagglutinin and neuraminidase membrane glycoproteins. J. Biol. Chem. 285, 28403–28409. doi: 10.1074/jbc.R110.129809, PMID: 20538598 PMC2937864

[ref46] Gao S. Song L. Li J. Zhang Z. Peng H. Jiang W.. (2012). Influenza A virus-encoded NS1 virulence factor protein inhibits innate immune response by targeting IKK. Cell. Microbiol. 14, 1849–1866. doi: 10.1111/cmi.12005, PMID: 22891964

[ref47] Garten W. Klenk H. D. (1999). Understanding influenza virus pathogenicity. Trends Microbiol. 7, 99–100. doi: 10.1016/S0966-842X(99)01460-2, PMID: 10203834

[ref48] Gaur P. Munjal A. Lal S. K. (2011). Influenza virus and cell signaling pathways. Med. Sci. Monit. 17, RA148–RA154. doi: 10.12659/MSM.881801, PMID: 21629204 PMC3539548

[ref49] Gómez-Puertas P. Albo C. Pérez-Pastrana E. Vivo A. Portela A. N. (2000). Influenza virus matrix protein is the major driving force in virus budding. J. Virol. 74, 11538–11547. doi: 10.1128/JVI.74.24.11538-11547.2000, PMID: 11090151 PMC112434

[ref50] Haasbach E. Pauli E. K. Spranger R. Mitzner D. Schubert U. Kircheis R.. (2011). Antiviral activity of the proteasome inhibitor VL-01 against influenza A viruses. Antiviral Res. 91, 304–313. doi: 10.1016/j.antiviral.2011.07.006, PMID: 21777621

[ref51] Hale B. G. Randall R. E. Ortin J. Jackson D. (2008). The multifunctional NS1 protein of influenza A viruses. J. Gen. Virol. 89, 2359–2376. doi: 10.1099/vir.0.2008/004606-0, PMID: 18796704

[ref52] Harris A. Forouhar F. Qiu S. Sha B. Luo M. (2001). The crystal structure of the influenza matrix protein M1 at neutral pH: M1-M1 protein interfaces can rotate in the oligomeric structures of M1. Virology 289, 34–44. doi: 10.1006/viro.2001.1119, PMID: 11601915

[ref53] Hayden F. G. Sugaya N. Hirotsu N. Lee N. De Jong M. D. Hurt A. C.. (2018). Baloxavir marboxil for uncomplicated influenza in adults and adolescents. N. Engl. J. Med. 379, 913–923. doi: 10.1056/NEJMoa1716197, PMID: 30184455

[ref54] Heaton N. S. Moshkina N. Fenouil R. Gardner T. J. Aguirre S. Shah P. S.. (2016). Targeting viral proteostasis limits influenza virus, HIV, and dengue virus infection. Immunity 44, 46–58. doi: 10.1016/j.immuni.2015.12.017, PMID: 26789921 PMC4878455

[ref55] Hebert D. N. Zhang J. X. Chen W. Foellmer B. Helenius A. (1997). The number and location of glycans on influenza hemagglutinin determine folding and association with calnexin and calreticulin. J. Cell Biol. 139, 613–623. doi: 10.1083/jcb.139.3.613, PMID: 9348279 PMC2141715

[ref56] Helenius A. (1992). Unpacking the incoming influenza virus. Cell 69, 577–578. doi: 10.1016/0092-8674(92)90219-3, PMID: 1375129

[ref57] Holsinger L. J. Alams R. (1991). Influenza virus M2 integral membrane protein is a homotetramer stabilized by formation of disulfide bonds. Virology 183, 32–43. doi: 10.1016/0042-6822(91)90115-R, PMID: 2053285

[ref58] Hu Y. Sneyd H. Dekant R. Wang J. (2017). Influenza A virus nucleoprotein: a highly conserved multi-functional viral protein as a hot antiviral drug target. Curr. Top. Med. Chem. 17, 2271–2285. doi: 10.2174/1568026617666170224122508, PMID: 28240183 PMC5967877

[ref59] Hu J. Zhang L. Liu X. (2020). Role of post-translational modifications in influenza A virus life cycle and host innate immune response. Front. Microbiol. 11:517461. doi: 10.3389/fmicb.2020.517461, PMID: 33013775 PMC7498822

[ref60] Husain M. Harrod K. S. (2011). Enhanced acetylation of alpha-tubulin in influenza A virus infected epithelial cells. FEBS Lett. 585, 128–132. doi: 10.1016/j.febslet.2010.11.023, PMID: 21094644

[ref61] Ito T. Couceiro J. N. Kelm S. Baum L. G. Krauss S. Castrucci M. R.. (1998). Molecular basis for the generation in pigs of influenza A viruses with pandemic potential. J. Virol. 72, 7367–7373. doi: 10.1128/JVI.72.9.7367-7373.1998, PMID: 9696833 PMC109961

[ref62] Ito T. Gorman O. T. Kawaoka Y. Bean W. J. Webster R. G. (1991). Evolutionary analysis of the influenza A virus M gene with comparison of the M1 and M2 proteins. J. Virol. 65, 5491–5498. doi: 10.1128/jvi.65.10.5491-5498.1991, PMID: 1895397 PMC249043

[ref63] Iwai A. Shiozaki T. Kawai T. Akira S. Kawaoka Y. Takada A.. (2010). Influenza A virus polymerase inhibits type I interferon induction by binding to interferon beta promoter stimulator 1. J. Biol. Chem. 285, 32064–32074. doi: 10.1074/jbc.M110.112458, PMID: 20699220 PMC2952208

[ref64] Jagger B. W. Wise H. M. Kash J. C. Walters K.-A. Wills N. M. Xiao Y.-L.. (2012). An overlapping protein-coding region in influenza A virus segment 3 modulates the host response. Science 337, 199–204. doi: 10.1126/science.1222213, PMID: 22745253 PMC3552242

[ref65] Jassal B. Matthews L. Viteri G. Gong C. Lorente P. Fabregat A.. (2020). The reactome pathway knowledgebase. Nucleic Acids Res. 48, D498–D503. doi: 10.1093/nar/gkz1031, PMID: 31691815 PMC7145712

[ref66] Jia D. Rahbar R. Chan R. W. Lee S. M. Chan M. C. Wang B. X.. (2010). Influenza virus non-structural protein 1 (NS1) disrupts interferon signaling. PLoS One 5:e13927. doi: 10.1371/journal.pone.0013927, PMID: 21085662 PMC2978095

[ref67] Karlas A. Machuy N. Shin Y. Pleissner K.-P. Artarini A. Heuer D.. (2010). Genome-wide RNAi screen identifies human host factors crucial for influenza virus replication. Nature 463, 818–822. doi: 10.1038/nature08760, PMID: 20081832

[ref68] Kido H. (2015). Influenza virus pathogenicity regulated by host cellular proteases, cytokines and metabolites, and its therapeutic options. Proc. Jpn. Acad. Ser. B Phys. Biol. Sci. 91, 351–368. doi: 10.2183/pjab.91.351, PMID: 26460316 PMC4729853

[ref69] Klemm C. Boergeling Y. Ludwig S. Ehrhardt C. (2018). Immunomodulatory nonstructural proteins of influenza A viruses. Trends Microbiol. 26, 624–636. doi: 10.1016/j.tim.2017.12.006, PMID: 29373257

[ref70] Kochs G. García-Sastre A. Martínez-Sobrido L. (2007). Multiple anti-interferon actions of the influenza A virus NS1 protein. J. Virol. 81, 7011–7021. doi: 10.1128/JVI.02581-06, PMID: 17442719 PMC1933316

[ref71] Koszalka P. Tilmanis D. Hurt A. C. (2017). Influenza antivirals currently in late-phase clinical trial. Influenza Other Respir. Viruses 11, 240–246. doi: 10.1111/irv.12446, PMID: 28146320 PMC5410715

[ref72] Krammer F. Smith G. J. D. Fouchier R. A. M. Peiris M. Kedzierska K. Doherty P. C.. (2018). Influenza. Nat. Rev. Dis. Primers 4:3. doi: 10.1038/s41572-018-0002-y, PMID: 29955068 PMC7097467

[ref73] Kuleshov M. V. Jones M. R. Rouillard A. D. Fernandez N. F. Duan Q. Wang Z.. (2016). Enrichr: a comprehensive gene set enrichment analysis web server 2016 update. Nucleic Acids Res. 44, W90–W97. doi: 10.1093/nar/gkw377, PMID: 27141961 PMC4987924

[ref74] Kummer S. Flottmann M. Schwanhausser B. Sieben C. Veit M. Selbach M.. (2014). Alteration of protein levels during influenza virus H1N1 infection in host cells: a proteomic survey of host and virus reveals differential dynamics. PLoS One 9:e94257. doi: 10.1371/journal.pone.0094257, PMID: 24718678 PMC3981805

[ref75] Kuo R. L. Krug R. M. (2009). Influenza a virus polymerase is an integral component of the CPSF30-NS1A protein complex in infected cells. J. Virol. 83, 1611–1616. doi: 10.1128/JVI.01491-08, PMID: 19052083 PMC2643760

[ref76] Lalime E. N. Pekosz A. (2014). The R35 residue of the influenza A virus NS1 protein has minimal effects on nuclear localization but alters virus replication through disrupting protein dimerization. Virology 458-459, 33–42. doi: 10.1016/j.virol.2014.04.012 24928037 PMC4095983

[ref77] Lamb R. A. Lai C. J. Choppin P. W. (1981). Sequences of mRNAs derived from genome RNA segment 7 of influenza virus: colinear and interrupted mRNAs code for overlapping proteins. Proc. Natl. Acad. Sci. U S A 78, 4170–4174. doi: 10.1073/pnas.78.7.4170, PMID: 6945577 PMC319750

[ref78] Lamb R. A. Zebedee S. L. Richardson C. D. (1985). Influenza virus M2 protein is an integral membrane protein expressed on the infected-cell surface. Cell 40, 627–633. doi: 10.1016/0092-8674(85)90211-9, PMID: 3882238

[ref79] Latham T. Galarza J. M. (2001). Formation of wild-type and chimeric influenza virus-like particles following simultaneous expression of only four structural proteins. J. Virol. 75, 6154–6165. doi: 10.1128/JVI.75.13.6154-6165.2001, PMID: 11390617 PMC114331

[ref80] Lebouder F. Morello E. Rimmelzwaan G. F. Bosse F. Péchoux C. Delmas B.. (2008). Annexin II incorporated into influenza virus particles supports virus replication by converting plasminogen into plasmin. J. Virol. 82, 6820–6828. doi: 10.1128/JVI.00246-08, PMID: 18448517 PMC2446977

[ref81] Lee J. H. Kim S. H. Pascua P. N. Song M. S. Baek Y. H. Jin X.. (2010). Direct interaction of cellular hnRNP-F and NS1 of influenza A virus accelerates viral replication by modulation of viral transcriptional activity and host gene expression. Virology 397, 89–99. doi: 10.1016/j.virol.2009.10.041, PMID: 19926108

[ref82] Li H. Bradley K. C. Long J. S. Frise R. Ashcroft J. W. Hartgroves L. C.. (2018). Internal genes of a highly pathogenic H5N1 influenza virus determine high viral replication in myeloid cells and severe outcome of infection in mice. PLOS Pathogens 14:e1006821. doi: 10.1371/journal.ppat.1006821, PMID: 29300777 PMC5771632

[ref83] Li B. Clohisey S. M. Chia B. S. Wang B. Cui A. Eisenhaure T.. (2020). Genome-wide CRISPR screen identifies host dependency factors for influenza A virus infection. Nat. Commun. 11:164. doi: 10.1038/s41467-020-19935-y, PMID: 31919360 PMC6952391

[ref84] Li S. Min J.-Y. Krug R. M. Sen G. C. (2006). Binding of the influenza A virus NS1 protein to PKR mediates the inhibition of its activation by either PACT or double-stranded RNA. Virology 349, 13–21. doi: 10.1016/j.virol.2006.01.005, PMID: 16466763

[ref85] Licatalosi D. D. Darnell R. B. (2010). RNA processing and its regulation: global insights into biological networks. Nat. Rev. Genet. 11, 75–87. doi: 10.1038/nrg2673, PMID: 20019688 PMC3229837

[ref86] Limsuwat N. Boonarkart C. Phakaratsakul S. Suptawiwat O. Auewarakul P. (2020). Influence of cellular lipid content on influenza A virus replication. Archives of Virology 165, 1151–1161. doi: 10.1007/s00705-020-04596-5, PMID: 32227307 PMC7223680

[ref87] Liu G. Xiang Y. Guo C. Pei Y. Wang Y. Kitazato K. (2014). Cofilin-1 is involved in regulation of actin reorganization during influenza A virus assembly and budding. Biochem. Biophys. Res. Commun. 453, 821–825. doi: 10.1016/j.bbrc.2014.10.036, PMID: 25450354

[ref88] Lou Z. Sun Y. Rao Z. (2014). Current progress in antiviral strategies. Trends in Pharmacological Sciences 35, 86–102. doi: 10.1016/j.tips.2013.11.006, PMID: 24439476 PMC7112804

[ref89] Ma Y. Sun J. Gu L. Bao H. Zhao Y. Shi L.. (2017). Annexin A2 (ANXA2) interacts with nonstructural protein 1 and promotes the replication of highly pathogenic H5N1 avian influenza virus. BMC Microbiol. 17:191. doi: 10.1186/s12866-017-1097-0, PMID: 28893180 PMC5594581

[ref90] Massari S. Goracci L. Desantis J. Tabarrini O. (2016). Polymerase acidic protein–basic protein 1 (PA–PB1) protein–protein interaction as a target for next-generation anti-influenza therapeutics. J. Med. Chem. 59, 7699–7718. doi: 10.1021/acs.jmedchem.5b01474, PMID: 27046062

[ref91] Mata M. A. Satterly N. Versteeg G. A. Frantz D. Wei S. Williams N.. (2011). Chemical inhibition of RNA viruses reveals REDD1 as a host defense factor. Nat. Chem. Biol. 7, 712–719. doi: 10.1038/nchembio.645, PMID: 21909097 PMC3329801

[ref92] Matrosovich M. N. Matrosovich T. Y. Gray T. Roberts N. A. Klenk H.-D. (2004). Neuraminidase is important for the initiation of influenza virus infection in human airway epithelium. J. Virol. 78, 12665–12667. doi: 10.1128/JVI.78.22.12665-12667.2004, PMID: 15507653 PMC525087

[ref93] Mccauley J. Hongo S. Kaverin N. Kochs G. Lamb R. Matrosovich M.. (2012). “Family – orthomyxoviridae,” in Virus Taxonomy. eds. King A. M. Q. Adams M. J. Carstens E. B. Lefkowitz E. J. (San Diego: Elsevier).

[ref94] MacLachlan N. J. Dubovi E. J. (2017). “Chapter 21 – Orthomyxoviridae,” in Fenner’s Veterinary Virology. 5th *Edn*. eds. Maclachlan N. J. Dubovi E. J. (Boston, MA: Academic Press).

[ref95] Murray R. Z. Stow J. L. (2014). Cytokine secretion in macrophages: SNAREs, Rabs, and membrane trafficking. Front. Immunol. 5:538. doi: 10.3389/fimmu.2014.00538, PMID: 25386181 PMC4209870

[ref96] Musiol A. Gran S. Ehrhardt C. Ludwig S. Grewal T. Gerke V.. (2013). Annexin A6-balanced late endosomal cholesterol controls influenza A replication and propagation. mBio 4:e00608-13. doi: 10.1128/mBio.00608-13, PMID: 24194536 PMC3892785

[ref97] Nailwal H. Sharma S. Mayank A. K. Lal S. K. (2015). The nucleoprotein of influenza A virus induces p53 signaling and apoptosis via attenuation of host ubiquitin ligase RNF43. Cell Death Dis. 6:e1768. doi: 10.1038/cddis.2015.131, PMID: 25996295 PMC4669709

[ref98] Nasser E. H. Judd A. K. Sanchez A. Anastasiou D. Bucher D. J. (1996). Antiviral activity of influenza virus M1 zinc finger peptides. J. Virol. 70, 8639–8644. doi: 10.1128/jvi.70.12.8639-8644.1996, PMID: 8970989 PMC190957

[ref99] Neumann G. Hughes M. T. Kawaoka Y. (2000). Influenza A virus NS2 protein mediates vRNP nuclear export through NES-independent interaction with hCRM1. EMBO J. 19, 6751–6758. doi: 10.1093/emboj/19.24.6751, PMID: 11118210 PMC305902

[ref100] Ng A. K.-L. Zhang H. Tan K. Li Z. Liu J.-H. Chan P. K.-S.. (2008). Structure of the influenza virus A H5N1 nucleoprotein: implications for RNA binding, oligomerization, and vaccine design. FASEB J. 22, 3638–3647. doi: 10.1096/fj.08-112110, PMID: 18614582 PMC2537428

[ref101] Noda T. (2012). Native morphology of influenza virions. Front. Microbiol. 2:269. doi: 10.3389/fmicb.2011.00269 22291683 PMC3249889

[ref102] Nogales A. Martinez-Sobrido L. Topham D. J. Dediego M. L. (2017). NS1 protein amino acid changes D189N and V194I affect interferon responses, thermosensitivity, and virulence of circulating H3N2 human influenza A viruses. J. Virol. 91:e01930-16. doi: 10.1128/JVI.01930-16, PMID: 28003482 PMC5309952

[ref103] O’neill R. E. Talon J. Palese P. (1998). The influenza virus NEP (NS2 protein) mediates the nuclear export of viral ribonucleoproteins. EMBO J. 17, 288–296. doi: 10.1093/emboj/17.1.288, PMID: 9427762 PMC1170379

[ref104] Ohno M. Sekiya T. Nomura N. Daito T. J. Shingai M. Kida H. (2020). Influenza virus infection affects insulin signaling, fatty acid-metabolizing enzyme expressions, and the tricarboxylic acid cycle in mice. Sci. Rep. 10:10879. doi: 10.1038/s41598-020-67879-6, PMID: 32616893 PMC7331672

[ref105] Ozawa M. Basnet S. Burley L. M. Neumann G. Hatta M. KAWAOKA Y. (2011). Impact of amino acid mutations in PB2, PB1-F2, and NS1 on the replication and pathogenicity of pandemic (H1N1) 2009 influenza viruses. J. Virol. 85, 4596–4601. doi: 10.1128/JVI.00029-11, PMID: 21325408 PMC3126221

[ref106] Paules C. I. Sullivan S. G. Subbarao K. Fauci A. S. (2018). Chasing seasonal influenza—the need for a universal influenza vaccine. N. Engl. J. Med. 378, 7–9. doi: 10.1056/NEJMp1714916, PMID: 29185857

[ref107] Pereira C. F. Read E. K. C. Wise H. M. Amorim M. J. Digard P. (2017). Influenza A virus NS1 protein promotes efficient nuclear export of unspliced viral M1 mRNA. J. Virol. 91:e00528-17. doi: 10.1128/JVI.00528-17, PMID: 28515301 PMC5651720

[ref108] Ramos I. Carnero E. Bernal-Rubio D. Seibert C. W. Westera L. García-Sastre A.. (2013). Contribution of double-stranded RNA and CPSF30 binding domains of influenza virus NS1 to the inhibition of type I interferon production and activation of human dendritic cells. J. Virol. 87, 2430–2440. doi: 10.1128/JVI.02247-12, PMID: 23255794 PMC3571370

[ref109] Ravindran M. S. Bagchi P. Cunningham C. N. Tsai B. (2016). Opportunistic intruders: how viruses orchestrate ER functions to infect cells. Nat. Rev. Microbiol. 14, 407–420. doi: 10.1038/nrmicro.2016.60, PMID: 27265768 PMC5272919

[ref110] Robb N. C. Smith M. Vreede F. T. Fodor E. (2009). NS2/NEP protein regulates transcription and replication of the influenza virus RNA genome. J. General Virol. 90, 1398–1407. doi: 10.1099/vir.0.009639-0, PMID: 19264657

[ref111] Rogers G. N. D’Souza B. L. (1989). Receptor binding properties of human and animal H1 influenza virus isolates. Virology 173, 317–322. doi: 10.1016/0042-6822(89)90249-3, PMID: 2815586

[ref112] Rogers G. N. Paulson J. C. (1983). Receptor determinants of human and animal influenza virus isolates: differences in receptor specificity of the H3 hemagglutinin based on species of origin. Virology 127, 361–373. doi: 10.1016/0042-6822(83)90150-2, PMID: 6868370

[ref113] Russ G. Bennink J. R. Bächi T. Yewdell J. W. (1991). Influenza virus hemagglutinin trimers and monomers maintain distinct biochemical modifications and intracellular distribution in brefeldin A-treated cells. Cell Regulation 2, 549–563. doi: 10.1091/mbc.2.7.549, PMID: 1664239 PMC361844

[ref114] Saito T. Tanaka M. Yamaguchi I. (1996). Effect of brefeldin A on influenza A virus-induced apoptosis in vitro. J. Vet. Med. Sci. 58, 1137–1139. doi: 10.1292/jvms.58.11_1137, PMID: 8959666

[ref115] Schaap I. A. T. Eghiaian F. Des Georges A. Veigel C. (2012). Effect of envelope proteins on the mechanical properties of influenza virus. The Journal of Biological Chemistry 287, 41078–41088. doi: 10.1074/jbc.M112.412726, PMID: 23048030 PMC3510809

[ref116] Sharma K. Tripathi S. Ranjan P. Kumar P. Garten R. Deyde V.. (2011). Influenza A virus nucleoprotein exploits Hsp40 to inhibit PKR activation. PLoS One 6:e20215. doi: 10.1371/journal.pone.0020215, PMID: 21698289 PMC3115951

[ref117] Shen Z. Lou K. Wang W. (2015). New small-molecule drug design strategies for fighting resistant influenza A. Acta Pharm. Sin. B 5, 419–430. doi: 10.1016/j.apsb.2015.07.006, PMID: 26579472 PMC4629447

[ref118] Short K. R. Kedzierska K. Van De Sandt C. E. (2018). Back to the future: lessons learned from the 1918 influenza pandemic. Front. Cell. Infect. Microbiol. 8:343. doi: 10.3389/fcimb.2018.00343, PMID: 30349811 PMC6187080

[ref119] Skehel J. J. Wiley D. C. (2000). Receptor binding and membrane fusion in virus entry: the influenza hemagglutinin. Annu. Rev. Biochem. 69, 531–569. doi: 10.1146/annurev.biochem.69.1.531, PMID: 10966468

[ref120] Steinhauer D. A. (1999). Role of hemagglutinin cleavage for the pathogenicity of influenza virus. Virology 258, 1–20. doi: 10.1006/viro.1999.9716, PMID: 10329563

[ref121] Stevaert A. Naesens L. (2016). The influenza virus polymerase complex: an update on its structure, functions, and significance for antiviral drug design. Med. Res. Rev. 36, 1127–1173. doi: 10.1002/med.21401, PMID: 27569399 PMC5108440

[ref122] Su W. C. Yu W. Y. Huang S. H. Lai M. M. C. (2018). Ubiquitination of the cytoplasmic domain of influenza A virus M2 protein is crucial for production of infectious virus particles. J. Virol. 92:e01972-1. doi: 10.1128/JVI.01972-17, PMID: 29167343 PMC5790949

[ref123] Sugiyama K. Obayashi E. Kawaguchi A. Suzuki Y. Tame J. R. H. Nagata K.. (2009). Structural insight into the essential PB1-PB2 subunit contact of the influenza virus RNA polymerase. EMBO J. 28, 1803–1811. doi: 10.1038/emboj.2009.138, PMID: 19461581 PMC2699363

[ref124] Sugrue R. J. Hay A. J. (1991). Structural characteristics of the M2 protein of influenza A viruses: evidence that it forms a tetrameric channel. Virology 180, 617–624. doi: 10.1016/0042-6822(91)90075-M, PMID: 1989386 PMC7131614

[ref125] Taguwa S. Maringer K. Li X. Bernal-Rubio D. Rauch J. N. Gestwicki J. E.. (2015). Defining Hsp70 subnetworks in dengue virus replication reveals key vulnerability in Flavivirus infection. Cell 163, 1108–1123. doi: 10.1016/j.cell.2015.10.046, PMID: 26582131 PMC4869517

[ref126] Te Velthuis A. J. W. Fodor E. (2016). Influenza virus RNA polymerase: insights into the mechanisms of viral RNA synthesis. Nat. Rev. Micro 14, 479–493. doi: 10.1038/nrmicro.2016.87, PMID: 27396566 PMC4966622

[ref127] Thompson M. G. Muñoz-Moreno R. Bhat P. Roytenberg R. Lindberg J. Gazzara M. R.. (2018). Co-regulatory activity of hnRNP K and NS1-BP in influenza and human mRNA splicing. Nat. Commun. 9:2407. doi: 10.1038/s41467-018-04779-4, PMID: 29921878 PMC6008300

[ref128] Tong S. Zhu X. Li Y. Shi M. Zhang J. Bourgeois M.. (2013). New world bats harbor diverse influenza A viruses. PLOS Pathog. 9:e1003657. doi: 10.1371/journal.ppat.1003657, PMID: 24130481 PMC3794996

[ref129] Tripathi S. Batra J. Cao W. Sharma K. Patel J. R. Ranjan P.. (2013). Influenza A virus nucleoprotein induces apoptosis in human airway epithelial cells: implications of a novel interaction between nucleoprotein and host protein Clusterin. Cell Death Dis. 4:e562. doi: 10.1038/cddis.2013.89, PMID: 23538443 PMC3615740

[ref130] Tripathi S. Pohl M. O. Zhou Y. Rodriguez-Frandsen A. Wang G. Stein D. A.. (2015). Meta- and orthogonal integration of influenza “OMICs” data defines a role for UBR4 in virus budding. Cell Host Microbe 18, 723–735. doi: 10.1016/j.chom.2015.11.002, PMID: 26651948 PMC4829074

[ref131] Twu K. Y. Noah D. L. Rao P. Kuo R.-L. Krug R. M. (2006). The CPSF30 binding site on the NS1A protein of influenza A virus is a potential antiviral target. J. Virol. 80, 3957–3965. doi: 10.1128/JVI.80.8.3957-3965.2006, PMID: 16571812 PMC1440456

[ref132] Varghese J. N. Laver W. G. Colman P. M. (1983). Structure of the influenza virus glycoprotein antigen neuraminidase at 2.9 A resolution. Nature 303, 35–40. doi: 10.1038/303035a0, PMID: 6843658

[ref133] Wakefield L. Brownlee G. G. (1989). RNA-binding properties of influenza A virus matrix protein M1. Nucleic Acids Res. 17, 8569–8580. doi: 10.1093/nar/17.21.8569, PMID: 2479906 PMC335028

[ref134] Wang X. Basler C. F. Williams B. R. Silverman R. H. Palese P. Garcia-Sastre A. (2002). Functional replacement of the carboxy-terminal two-thirds of the influenza A virus NS1 protein with short heterologous dimerization domains. J. Virol. 76, 12951–12962. doi: 10.1128/JVI.76.24.12951-12962.2002, PMID: 12438621 PMC136679

[ref135] Watanabe T. Kawakami E. Shoemaker J. E. Lopes T. J. Matsuoka Y. Tomita Y.. (2014). Influenza virus-host interactome screen as a platform for antiviral drug development. Cell Host Microbe 16, 795–805. doi: 10.1016/j.chom.2014.11.002, PMID: 25464832 PMC4451456

[ref136] Watanabe T. Watanabe S. Kawaoka Y. (2010). Cellular networks involved in the influenza virus life cycle. Cell Host Microbe 7, 427–439. doi: 10.1016/j.chom.2010.05.008, PMID: 20542247 PMC3167038

[ref137] Wilkinson M. E. Charenton C. Nagai K. (2020). RNA splicing by the spliceosome. Ann. Rev. Biochem. 89, 359–388. doi: 10.1146/annurev-biochem-091719-064225, PMID: 31794245

[ref138] Wolff T. O’neill R. E. Palese P. (1998). NS1-binding protein (NS1-BP): a novel human protein that interacts with the influenza A virus nonstructural NS1 protein is relocalized in the nuclei of infected cells. J. Virol. 72, 7170–7180. doi: 10.1128/JVI.72.9.7170-7180.1998, PMID: 9696811 PMC109939

[ref139] Xia C. Vijayan M. Pritzl C. J. Fuchs S. Y. Mcdermott A. B. Hahm B.. (2016). Hemagglutinin of influenza A virus antagonizes type I interferon (IFN) responses by inducing degradation of type I IFN receptor 1. J. Virol. 90, 2403–2417. doi: 10.1128/JVI.02749-15, PMID: 26676772 PMC4810695

[ref140] Yadav V. Panganiban A. T. Honer Zu Bentrup K. Voss T. G. (2016). Influenza infection modulates vesicular trafficking and induces Golgi complex disruption. Virusdisease 27, 357–368. doi: 10.1007/s13337-016-0347-3, PMID: 28004015 PMC5142599

[ref141] Yasuda J. Nakada S. Kato A. Toyoda T. Ishihama A. (1993). Molecular assembly of influenza virus: association of the NS2 protein with virion matrix. Virology 196, 249–255. doi: 10.1006/viro.1993.1473, PMID: 8356796

[ref142] Ye Q. Krug R. M. Tao Y. J. (2006). The mechanism by which influenza A virus nucleoprotein forms oligomers and binds RNA. Nature 444, 1078–1082. doi: 10.1038/nature05379, PMID: 17151603

[ref143] Zhang J. Ruan T. Sheng T. Wang J. Sun J. Wang J.. (2019). Role of c-Jun terminal kinase (JNK) activation in influenza A virus-induced autophagy and replication. Virology 526, 1–12. doi: 10.1016/j.virol.2018.09.020, PMID: 30316042 PMC6424123

[ref144] Zhang K. Shang G. Padavannil A. Wang J. Sakthivel R. Chen X.. (2018). Structural–functional interactions of NS1-BP protein with the splicing and mRNA export machineries for viral and host gene expression. Proc. Nat. Acad. Sci. 115, E12218–E12227. doi: 10.1073/pnas.1818012115, PMID: 30538201 PMC6310826

[ref145] Zhirnov O. P. Klenk H. D. (2013). Influenza A virus proteins NS1 and hemagglutinin along with M2 are involved in stimulation of autophagy in infected cells. J. Virol. 87, 13107–13114. doi: 10.1128/JVI.02148-13, PMID: 24027311 PMC3838240

