# Supplementary material for: A Review and Meta-Analysis of Influenza Interactome Studies
Source: Front Microbiol. 2022 Apr 21;13:869406. doi: 10.3389/fmicb.2022.869406 (PMC9069142; doi:10.3389/fmicb.2022.869406)
Supplement: Supplementary file 2 [file Data_Sheet_1.pdf]

Supplementary Material

Supplementary Table 1: Summary of the materials and methodologies of used references in this study

Cell Line	Viral strain	Method	Reference
Primary swine respiratory epithelial cells (SRECs)	A/Sw/SK/18789/02 (H1N1) with Strep tagged NS gene	IP followed by LC-MS/MS	[1]
Y187, AH109	A/Puerto Rico/8/34 (H1N1), A/WSN/1933 TS61 (H1N1), A/Chicken/Scotland/1959 (H5N1), A/Chicken/Kurgan/3/2005 (H5N1), A/Vietnam/1194/2004 (H5N1), A/Chicken/Belgium/03 (H7N7), A/Equine/Prague/1/1956 (H7N7), A/Chicken/Guangdong/6/97 (H9N2), A/Duck/Australia/348/83 (H15N8)	Yeast 2 hybrid using human spleen, fetal brain and respiratory epithelium Gal4 AD-cDNA libraries in AH109 as the prey strain and Influenza ORFs in bait vector for Y187	[2]
Human telomerase reverse transcriptase-immortalized retinal pigment (RPE) cells	Influenza A/WSN/33(H1N1) viruses expressing wild type (WSNWT) or R38A/K41A mutant NS1 (WSNRK/AA)	ChIP followed by mass spectrometery	[3]
HEK293T	Influenza A virus Puerto Rico/1934/H1N1 strain (PR8)	IP with NS1 followed by Mass spectrometry	[4]
HEK293T	A/Taiwan/1/2013(H7N9)	IP with NS1 followed by Mass spectrometry	[5]
HEK293	Influenza A/Puerto Rico/8/34 (H1N1), influenza A/WSN/33 (H1N1), influenza A virus A/New York/18/2009 (H1N1) pdm09, and A/Aichi/68 (H3N2), VN/2004, A/ /Viet Nam/1203/2004(H5N1)	IP followed by Mass spectrometry	[6]
A549	A/WSN/33 (H1N1)	RNA immunoprecipitation followed by RNA sequencing	[7]

A549	Influenza A virus Puerto Rico/1934/H1N1 strain (PR8)	IP followed by Mass spectrometry	[8]
A549, HEK293T (SILAC-MS)	Influenza A virus Puerto Rico/1934/H1N1 strain (PR8)	TAP followed by Mass spectrometry, SILAC mass spectrometry used for quantification	[9]
None	3F5T PDB	Computer based model using structural homology and interaction redundacy	[10]
MATα Y8930	A/Udorn/72 (H3N2) and Influenza A/PR/8/34 (H1N1)	Yeast 2 hybrid using Human ORFeome v3.1 as prey and Genome wide RNAi screen	[11]
A549	A/WSN/33 w ith HA sequence substituted by Renilla	Genome Wide RNAi screen	[12]
A549	PR8 virus containing FLAG-tagged viral proteins	IP followed by Mass spectrometry	[13]
HEK293T	WSN	IP followed by Mass spectrometry with RNAi screen	[14]

A549	H1N1 (A/WSN/33) and H5N1 (A/Vietnam/1203/04)	Co-IP followed by Mass Spectrometry	[15]
HEK293T	H7N9 (A/shanghai/1/2013)	Co-IP followed by Mass Spectrometry	[16]
Nil	51,242 IAV sequences (H1N1, H3N2, H1N2, and H5N1)	Bioinformatics identification of human motif-domain interactions	[17]

Supplementary Figures

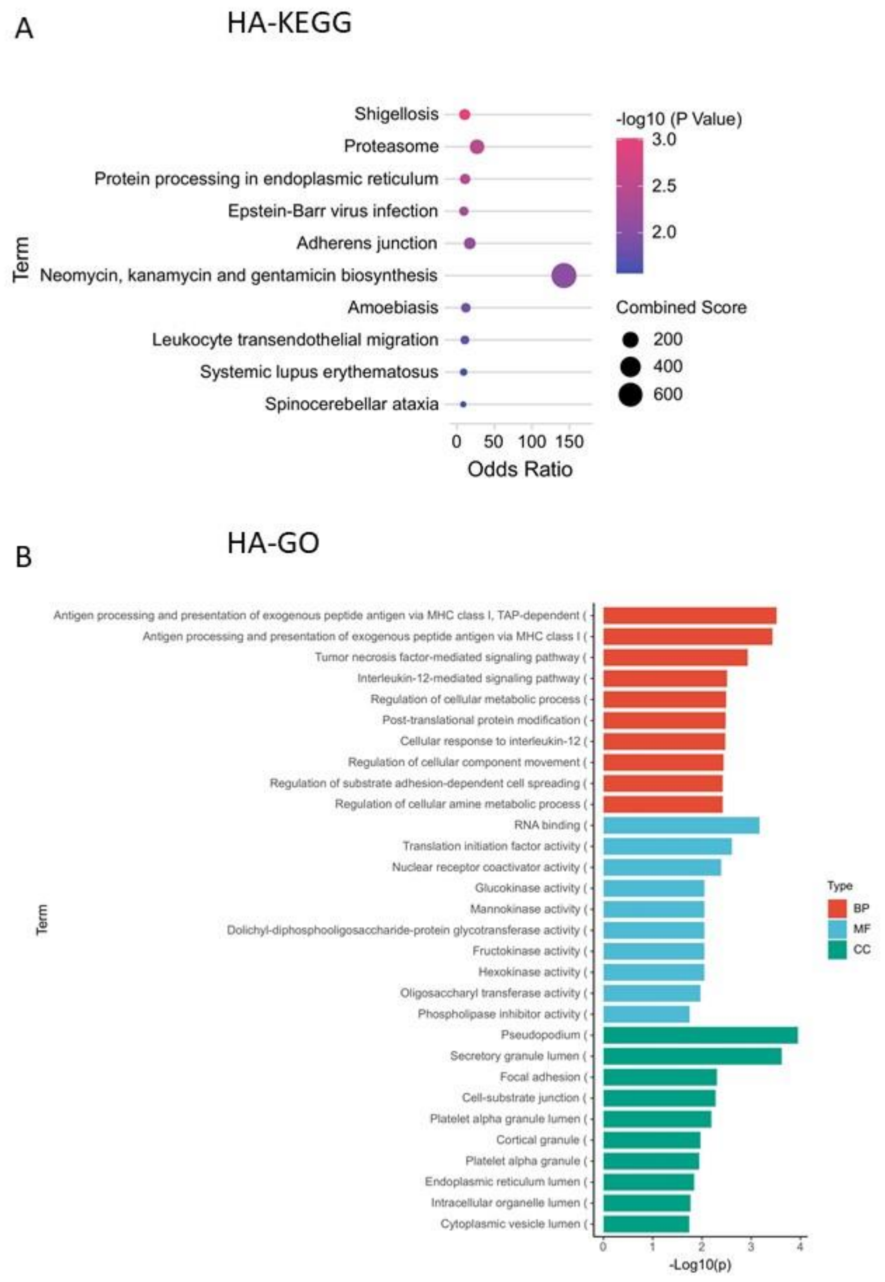

Figure S1 KEGG and Enrichr analysis of HA interactors. (A) Enriched KEGG pathway for HA interactors (B) GO analysis of HA interactors. Proteins were analyzed using Enrichr BP, MF, and CC represent Biological Process, Molecular Function, and Cellular Component groups of gene ontology (GO).

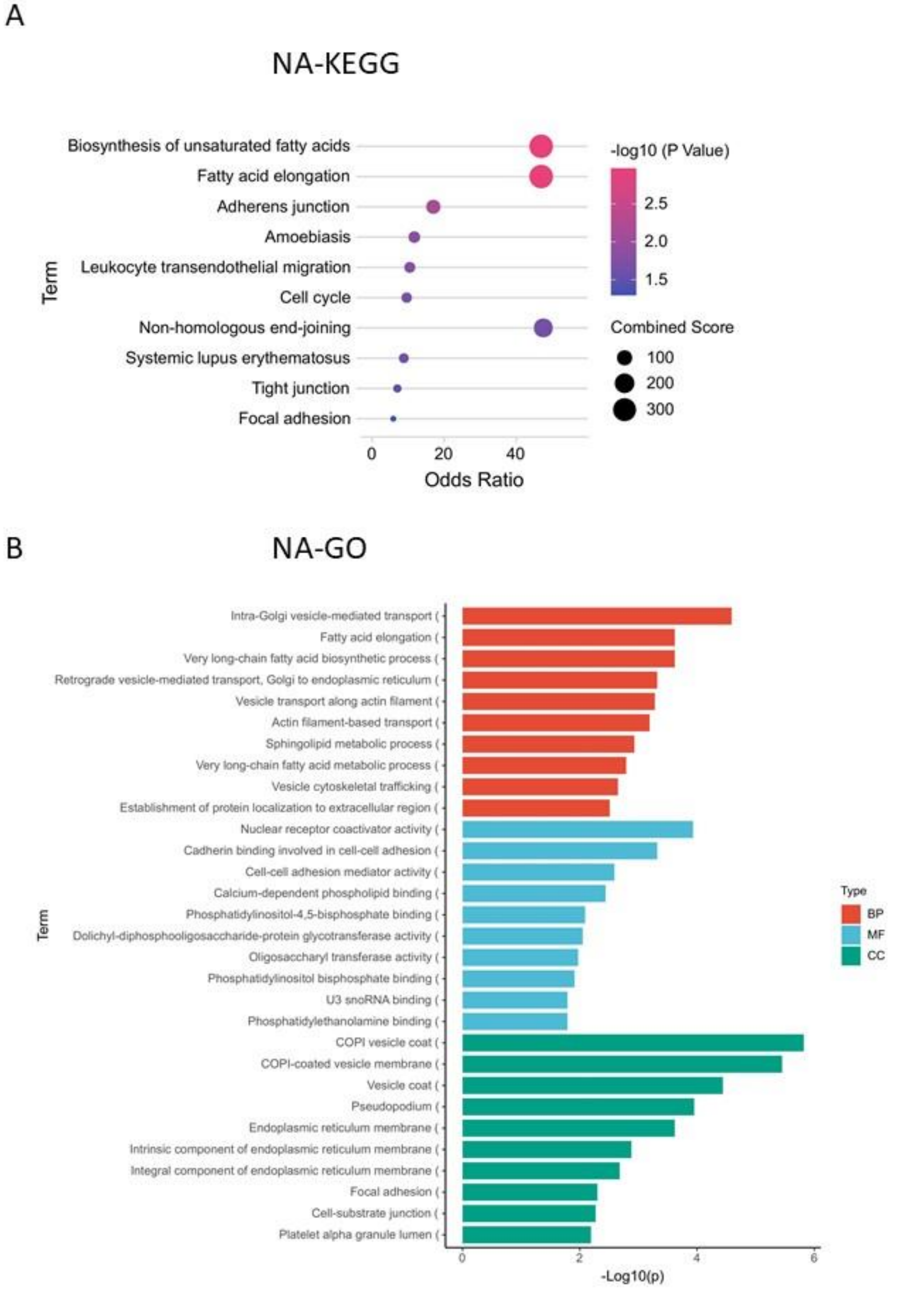

Fig S 2 KEGG and Enrichr analysis of NA interactors (A) Enriched KEGG pathway for NA interactors (B) GO analysis of NA interactors. Proteins were analyzed using Enrichr. BP, MF, and CC represent Biological Process, Molecular Function, and Cellular Component groups of gene ontology (GO).

A

NP-KEGG

B

PA-KEGG

C

PB1/2-KEGG

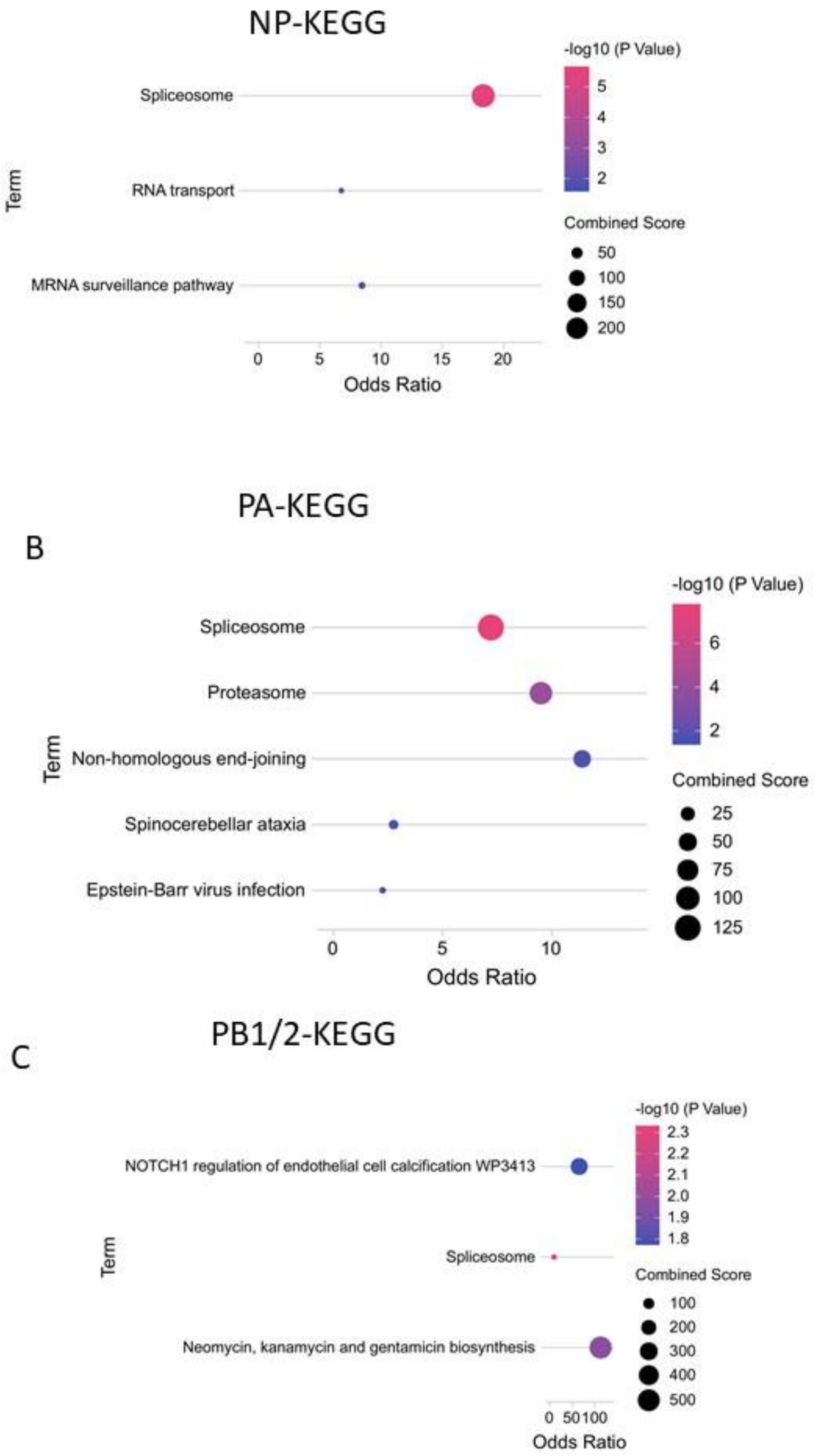

D NP-GO

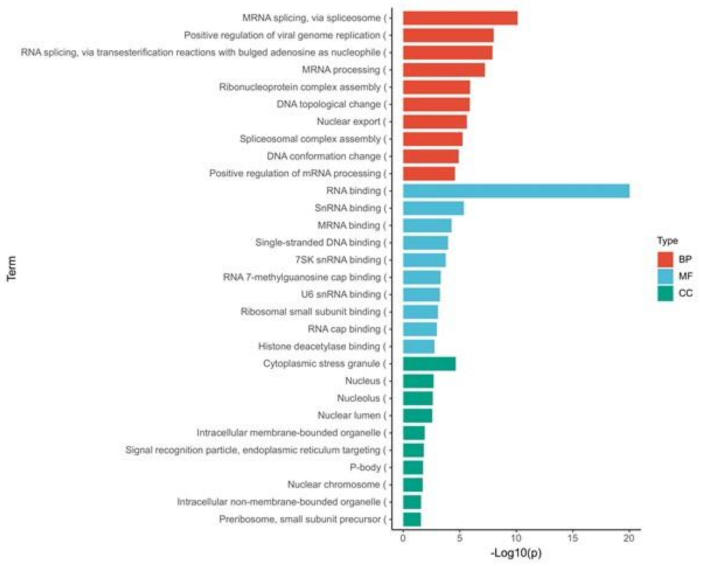
MRNA splicing, via spliceosome (
Positive regulation of viral genome replication (
RNA splicing, via transesterification reactions with bulged adenosine as nucleophile (
MRNA processing (
Ribonucleoprotein complex assembly (
DNA topological change (
Nuclear export (
Spliceosomal complex assembly (
DNA conformation change (
Positive regulation of mRNA processing (
RNA binding (
SnRNA binding (
MRNA binding (
Single-stranded DNA binding (
7SK snRNA binding (
RNA 7-methylguanosine cap binding (
U6 snRNA binding (
Ribosomal small subunit binding (
RNA cap binding (
Histone deacetylase binding (
Cytoplasmic stress granule (
Nucleus (
Nucleolus (
Nuclear lumen (
Intracellular membrane-bounded organelle (
Signal recognition particle, endoplasmic reticulum targeting (
P-body (
Nuclear chromosome (
Intracellular non-membrane-bounded organelle (
Preribosome, small subunit precursor (
Term
0
5
10
15
20
-Log10(p)
Type
BP
MF
CC

E PA-KEGG

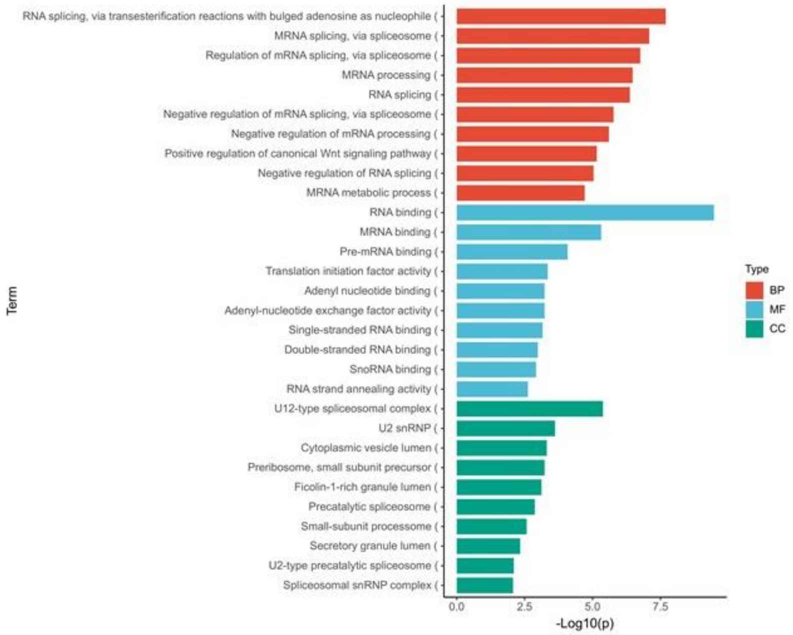

F PB1 GO

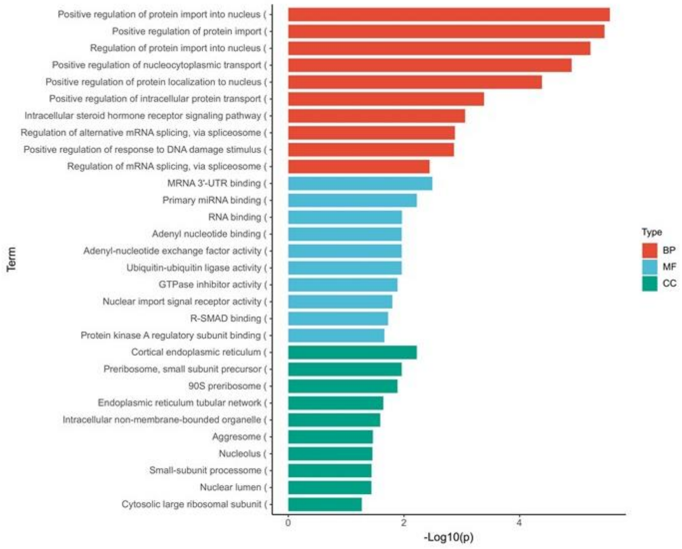

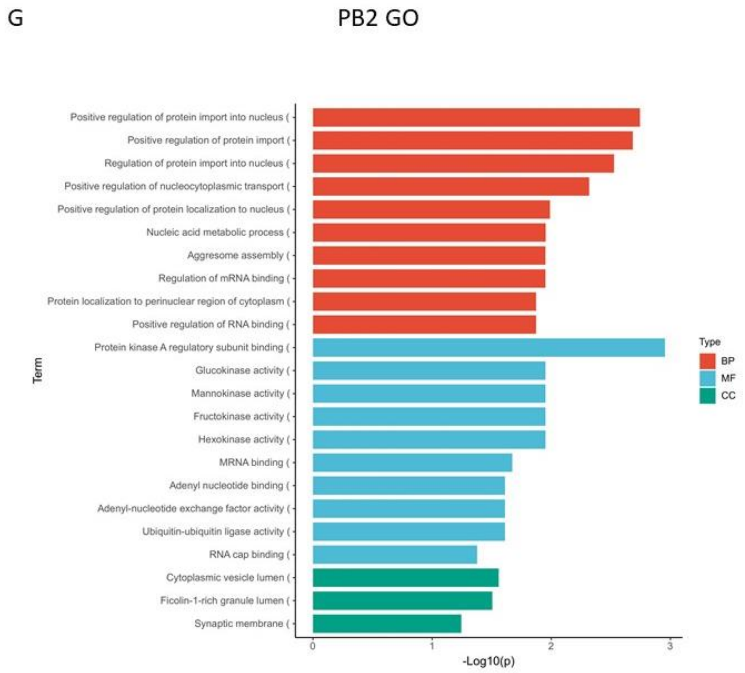

Fig S3 KEGG and Enrichr analysis of NP, PA, PB1, PB2 interactors (A) Enriched KEGG pathway for NP interactors (B) Enriched GO for PA interactors (C) Enriched KEGG pathway for PB1/PB2 interactors (D) GO analysis of NP interactors. (E) Functional enrichment analysis of PA interactors. (F) GO analysis of PB1 interactors. (G) GO analysis of NP interactors. Proteins were analyzed using Enrichr. BP, MF, and CC represent Biological Process, Molecular Function, and Cellular Component groups of gene ontology (GO).

A M1-KEGG

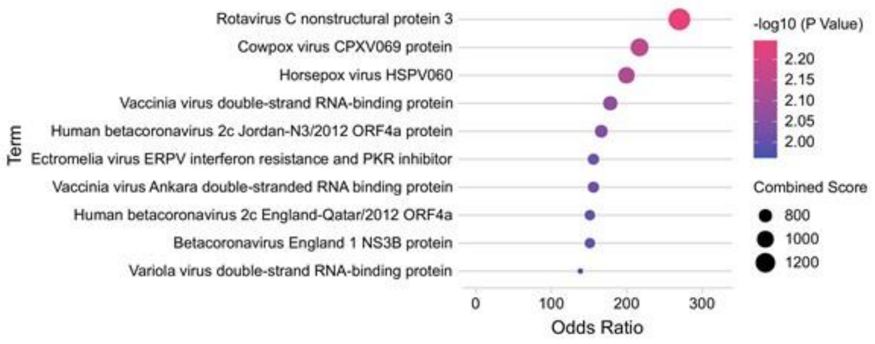

B M1-GO

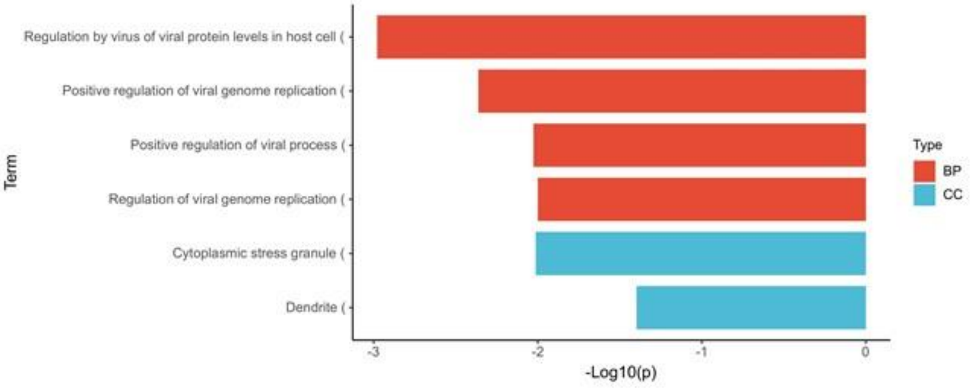

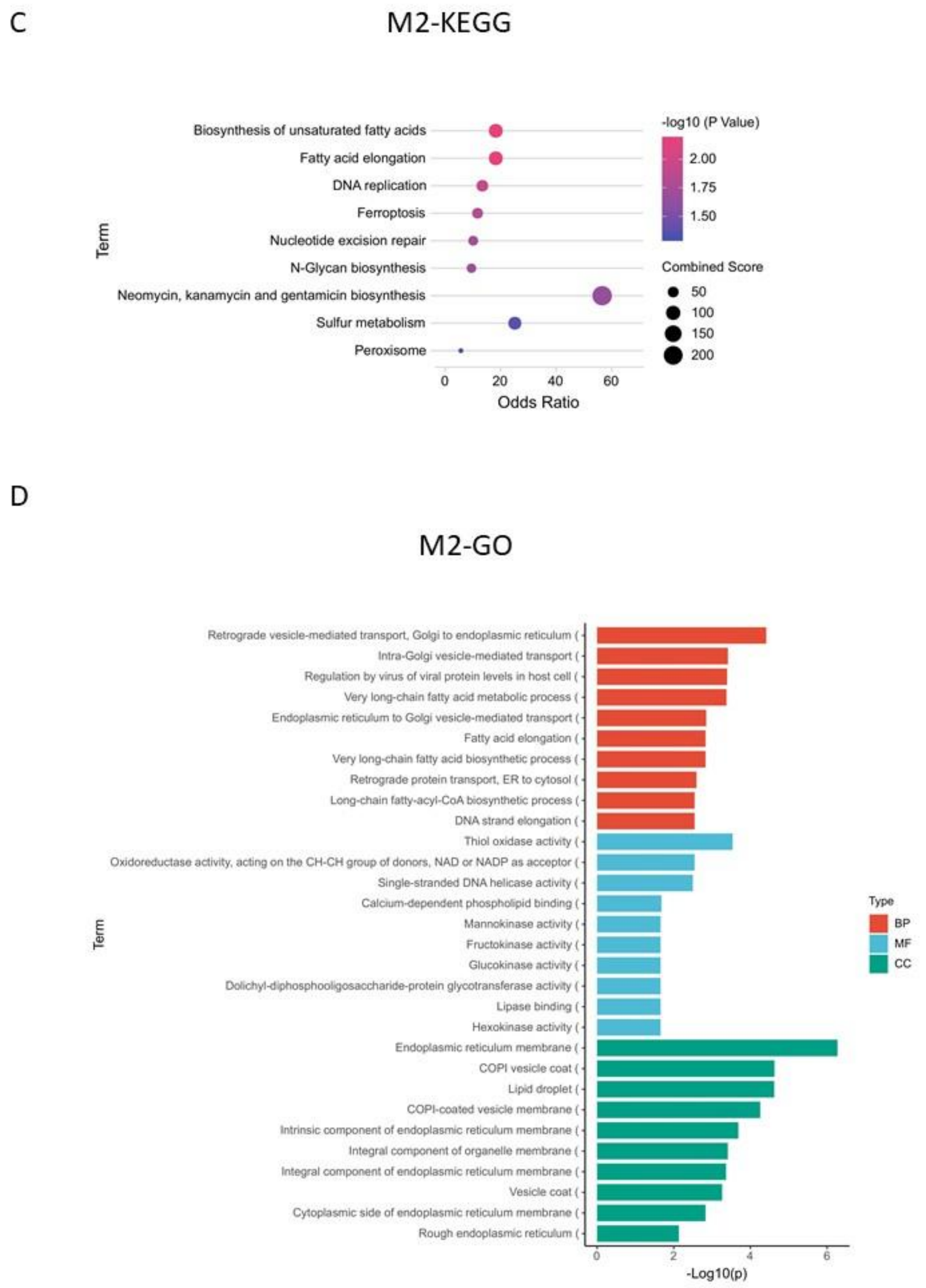

Fig S4 KEGG and Enrichr analysis of M1 and M2 interactors (A) Enriched KEGG pathway for M1 interactors (B) GO analysis of M1 interactors. Proteins were analyzed using Enrichr. (C) Enriched KEGG pathway for M2 interactors (D) GO analysis of M2 interactors. Proteins were analyzed using Enrichr. BP, MF, and CC represent Biological Process, Molecular Function, and Cellular Component groups of gene ontology (GO).

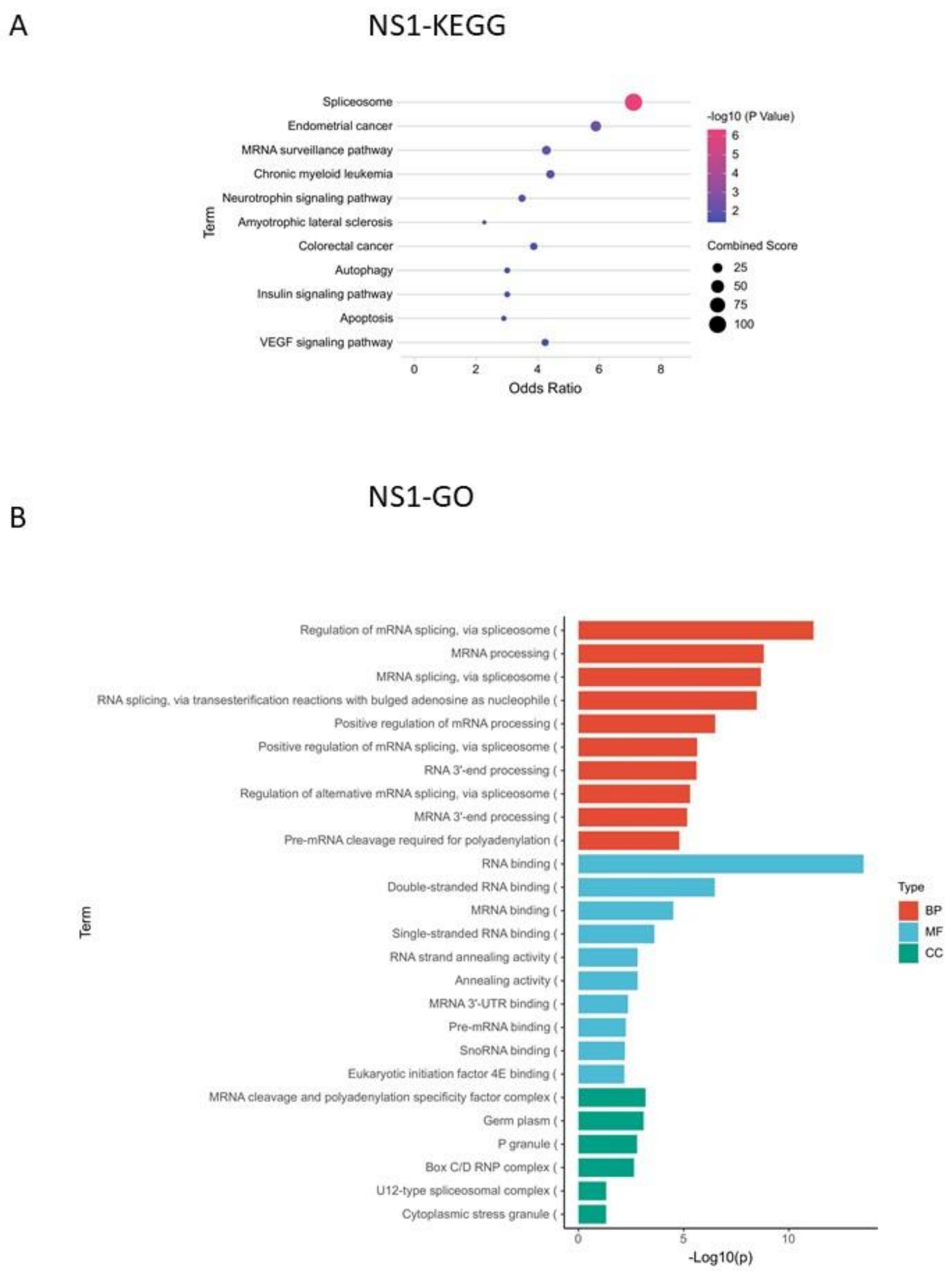

Fig S5 KEGG and Enrichr analysis of NS1 interactors (A) Enriched KEGG pathway for NS1 interactors (B) GO analysis of NS1 interactors. Proteins were analyzed using Enrichr. BP, MF, and CC represent Biological Process, Molecular Function, and Cellular Component groups of gene ontology (GO).

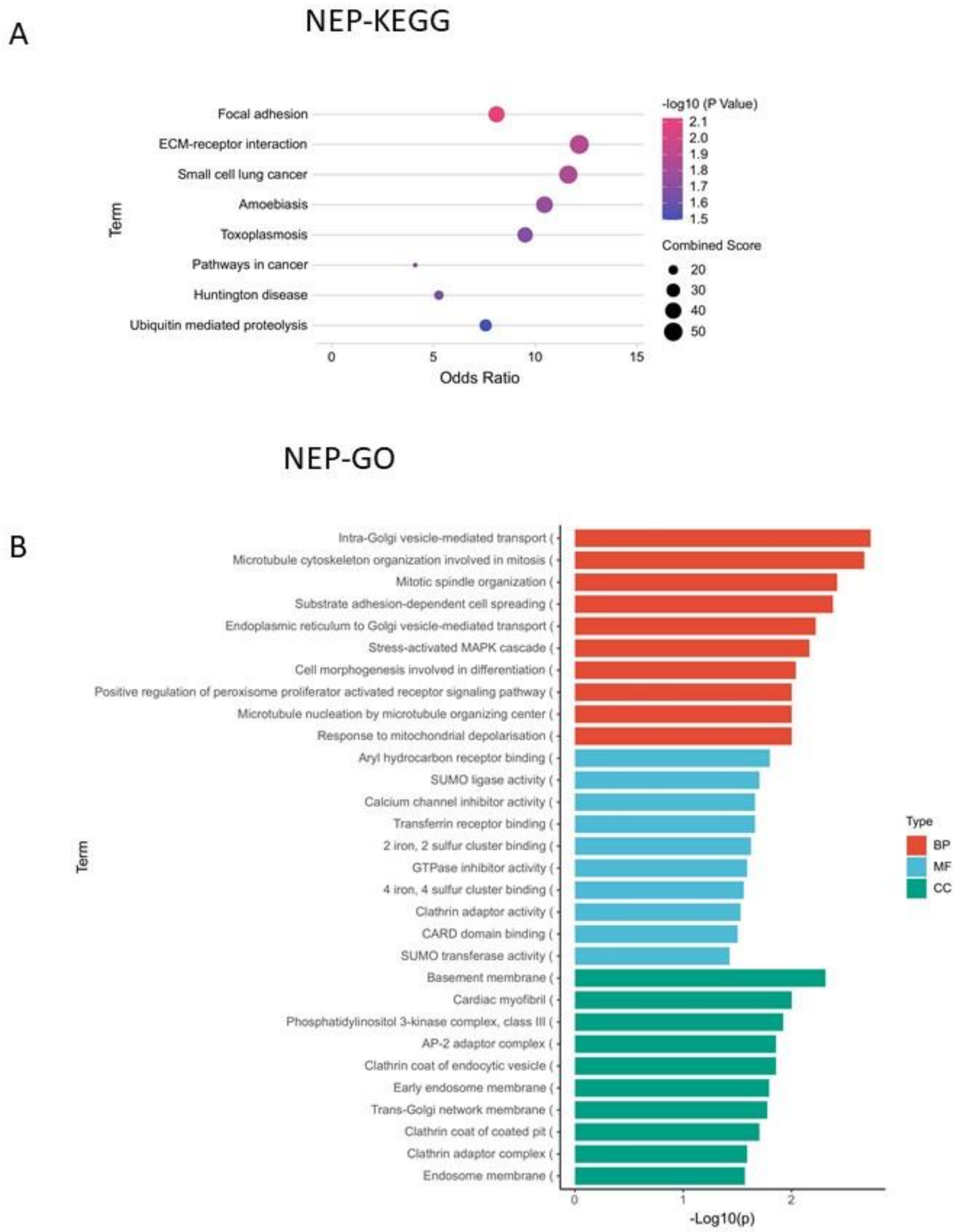

Fig S6 KEGG and Enrichr analysis of NEP interactors (A) Enriched KEGG pathway for NEP interactors (B) GO analysis of NEP interactors. Proteins were analyzed using Enrichr. BP, MF, and CC represent Biological Process, Molecular Function, and Cellular Component groups of gene ontology (GO).

References

1. Thulasi Raman, S.N. and Y. Zhou, *Networks of Host Factors that Interact with NS1 Protein of Influenza A Virus.* Frontiers in Microbiology, 2016. **7**: p. 654.

2. de Chassey, B., et al., *The interactomes of influenza virus NS1 and NS2 proteins identify new host factors and provide insights for ADAR1 playing a supportive role in virus replication.* PLoS pathogens, 2013. **9**(7): p. e1003440-e1003440.

3. Anastasina, M., et al., *Influenza virus NS1 protein binds cellular DNA to block transcription of antiviral genes.* Biochimica et Biophysica Acta (BBA) - Gene Regulatory Mechanisms, 2016. **1859**(11): p. 1440-1448.

4. Kuo, R.L., et al., *Interactome Analysis of the NS1 Protein Encoded by Influenza A H1N1 Virus Reveals a Positive Regulatory Role of Host Protein PRP19 in Viral Replication.* J Proteome Res, 2016. **15**(5): p. 1639-48.

5. Kuo, R.-L., et al., *Interactome Analysis of NS1 Protein Encoded by Influenza A H7N9 Virus Reveals an Inhibitory Role of NS1 in Host mRNA Maturation.* Journal of Proteome Research, 2018. **17**(4): p. 1474-1484.

6. Wang, L., et al., *Comparative influenza protein interactomes identify the role of plakophilin 2 in virus restriction.* Nature Communications, 2017. **8**: p. 13876.

7. Zhang, L., et al., *Influenza Virus NS1 Protein-RNA Interactome Reveals Intron Targeting.* Journal of Virology, 2018. **92**(24): p. e01634-18.

8. Rahim, M.N., et al., *Global Interactomics Connect Nuclear Mitotic Apparatus Protein NUMA1 to Influenza Virus Maturation.* Viruses, 2018. **10**(12): p. 731.

9. Tawaratsumida, K., et al., *Quantitative proteomic analysis of the influenza A virus nonstructural proteins NS1 and NS2 during natural cell infection identifies PACT as an NS1 target protein and antiviral host factor.* J Virol, 2014. **88**(16): p. 9038-48.

10. de Chassey, B., et al., *Structure homology and interaction redundancy for discovering virus-host protein interactions.* EMBO reports, 2013. **14**(10): p. 938-944.

11. Shapira, S.D., et al., *A physical and regulatory map of host-influenza interactions reveals pathways in H1N1 infection.* Cell, 2009. **139**(7): p. 1255-67.

12. König, R., et al., *Human host factors required for influenza virus replication.* Nature, 2010. **463**(7282): p. 813-817.

13. Heaton, N.S., et al., *Targeting Viral Proteostasis Limits Influenza Virus, HIV, and Dengue Virus Infection.* Immunity, 2016. **44**(1): p. 46-58.

14. Watanabe, T., et al., *Influenza virus-host interactome screen as a platform for antiviral drug development.* Cell Host Microbe, 2014. **16**(6): p. 795-805.

15. Bradel-Tretheway, B.G., et al., *Comprehensive proteomic analysis of influenza virus polymerase complex reveals a novel association with mitochondrial proteins and RNA polymerase accessory factors.* J Virol, 2011. **85**(17): p. 8569-81.

16. Sun, N., et al., *Proteomics Analysis of Cellular Proteins Co-Immunoprecipitated with Nucleoprotein of Influenza A Virus (H7N9).* Int J Mol Sci, 2015. **16**(11): p. 25982-98.

17. García-Pérez, C.A., et al., *Proteome-wide analysis of human motif-domain interactions mapped on influenza a virus.* BMC bioinformatics, 2018. **19**(1): p. 238-238.
